# A Comprehensive Characterization of the Function of LincRNAs in Transcriptional Regulation Through Long-Range Chromatin Interactions

**DOI:** 10.1038/srep36572

**Published:** 2016-11-08

**Authors:** Liuyang Cai, Huidan Chang, Yaping Fang, Guoliang Li

**Affiliations:** 1 National Key Laboratory of Crop Genetic Improvement, Agricultural Bioinformatics Key Laboratory of Hubei Province, College of Informatics, Huazhong Agricultural University, Wuhan, Hubei 430070, China

## Abstract

LincRNAs are emerging as important regulators with various cellular functions. However, the mechanisms behind their role in transcriptional regulation have not yet been fully explored. In this report, we proposed to characterize the diverse functions of lincRNAs in transcription regulation through an examination of their long-range chromatin interactions. We found that the promoter regions of lincRNAs displayed two distinct patterns of chromatin states, promoter-like and enhancer-like, indicating different regulatory functions for lincRNAs. Notably, the chromatin interactions between lincRNA genes and other genes suggested a potential mechanism for lincRNAs in the regulation of other genes at the RNA level because the transcribed lincRNAs could function at local spaces on other genes that interact with the lincRNAs at the DNA level. These results represent a novel way to predict the functions of lincRNAs. The GWAS-identification of SNPs within the lincRNAs revealed that some lincRNAs were disease-associated, and the chromatin interactions with those lincRNAs suggested that they were potential target genes of these lincRNA-associated SNPs. Our study provides new insights into the roles that lincRNAs play in transcription regulation.

Long noncoding RNAs (lncRNAs) are transcribed from the non-coding portions of the genome. They contain more than 200 nucleotides with little or no coding potential, although new evidence has suggested that lncRNAs can be translated to peptides^1^. Recent studies have shown that lncRNAs play important roles in transcription regulation, epigenetic regulation, and development^2^,^3^,^4^. Projects such as GENCODE^5^ have annotated an extensive catalog of lncRNAs in the human and mouse genome. However, the properties of most lncRNAs and their functions are not well characterized.

Long intergenic noncoding RNAs (lincRNAs) are a class of lncRNAs that do not overlap with the bodies of known protein-coding genes. This study primarily focuses on lincRNAs because the lack of overlap with protein-coding genes results in fewer complications in experiments and data analysis. Analysis has revealed that some specific lincRNAs have functions at the molecular and cellular levels. For example, the lincRNA MALAT1 (Metastasis Associated Lung Adenocarcinoma Transcript 1) regulates the expression of metastasis-associated genes^6^ and alternative splicing^7^. Another lincRNA NEAT1 (Nuclear Enriched Abundant Transcript 1) is an essential component of paraspeckles^8^.

Recent studies have indicated that there is a link between lincRNA function and genome spatial organization. For example, the lincRNA Firre colocalizes with its *trans* target genes^9^, and the lincRNA CCAT1-L maintains long-range interactions between MYC and its enhancers^10^. These results suggest that genome spatial organization may play a role in the functions of lincRNAs. In addition, lincRNAs can also impact nuclear structure^11^.

In recent years, technologies derived from the Chromosome Conformation Capture(3C)^12^ method have shown that the spatial organization of genome and chromatin interactions play key roles in transcription regulation^13^,^14^,^15^. Chromatin Interaction Analysis with Paired-End Tag (ChIA-PET) sequencing is a 3C-derived technology^16^ that can be used to explore chromatin interactions mediated by specific proteins and has been applied to a number of human and mouse cell lines^17^,^18^,^19^ (see ref. 20 for a review). Genome-wide chromatin interaction data captured by ChIA-PET sequencing can be analyzed using a network approach^21^. Among them, RNA polymerase II (RNAPII)-associated ChIA-PET data identify the chromatin interactome associated with transcription regulation. Previous studies have investigated the relationships between the interactome and the transcription regulation of protein-coding genes^17^,^21^ and miRNA genes^22^. Because most lincRNAs are transcribed by RNAPII, they are also components of the chromatin interaction network and could be studied using the network approach.

In this study, we characterized lincRNAs by examining long-range chromatin interactions. We examined the chromatin interaction data from two human cell lines and four mouse cell lines and integrated the extra data, including the transcriptome RNA-Seq data and the histone modification ChIP-Seq data, to annotate the chromatin interactions of the lincRNAs to establish a link between the higher-order chromatin organizations and the functions of lincRNAs in transcription. We primarily focused on the RNAPII-associated ChIA-PET data from the K562 and MCF7 cell lines^15^ but also used data from the other four cell lines to display specific examples.

## Results

### Transcription-associated chromatin interaction networks involving non-coding RNAs and protein-coding genes

In this study, we used RNAPII-mediated ChIA-PET data to construct transcription-associated chromatin interaction networks (termed as ChINs^21^), which were originally described by Li *et al*. in 2012^17^. In these networks, the nodes represent the genomic regions involved in chromatin interactions, and the edges represent the chromatin interactions between the different genomic regions.

**Figure 1:**
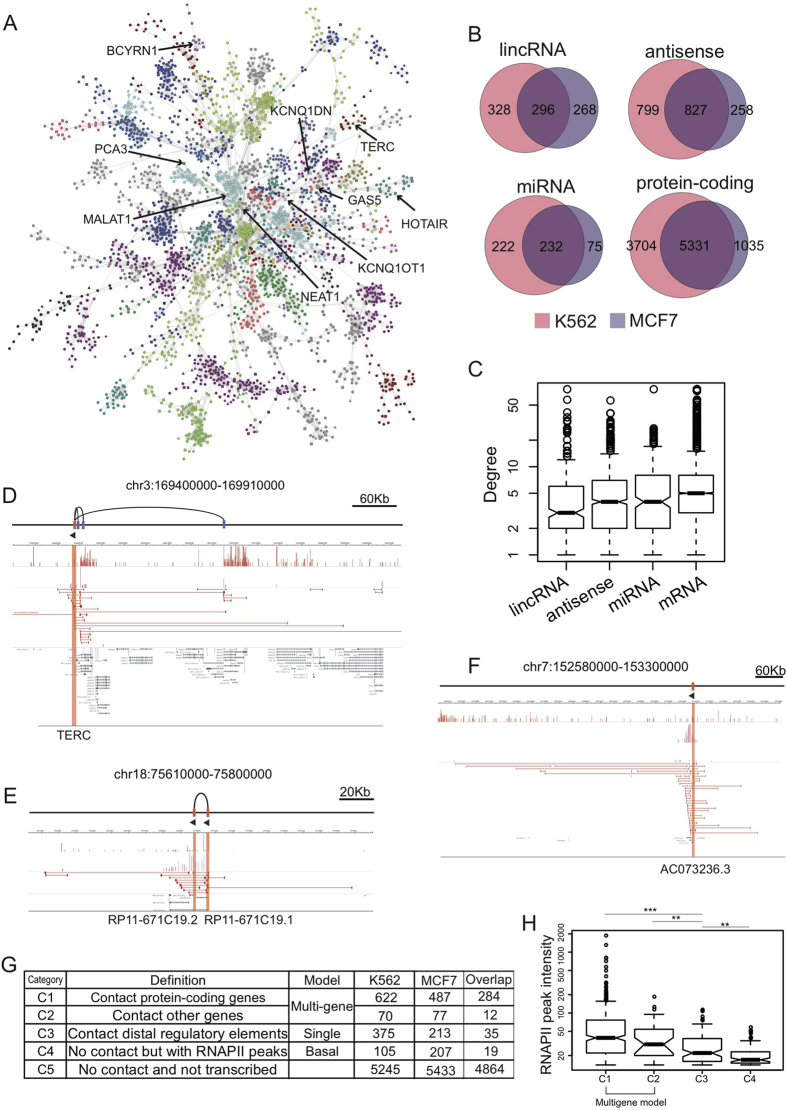
Chromatin Interaction Networks (ChINs) involving non-coding RNAs and protein-coding genes in K562. (**A**) The largest sub-network (as giant component) of ChIN. The different colors of the nodes represent different chromosomes (refer to Supplementary Table S3). Certain known lncRNAs are labeled with arrows. (**B**) Venn diagrams of the different types of genes in the ChINs of the K562 and MCF7 cells. (**C**) Box plots of degrees from the different types of genes in the ChIN. (**D–F**) Examples of lincRNA genes in categories C1–C3. (**D**) Category C1 (TERC interacts with some protein-coding genes), (**E**) C2 (RP11-671C19.1 interacts with lincRNA genes other than protein-coding genes) and (**F**) C3 (AC073236.3 interacts with non-promoter elements). Categories C1–C5 are defined in (**G**). (**G**) Definitions of the different categories of lincRNAs (C1–C5) and the numbers of lincRNAs in each category. All of the overlaps of the five categories are significant. (p-value < 0.001, Fisher Exact Test). (**H**). RNAPII signal intensities (log2 transformed) of lincRNA genes in different interaction categories.

We first examined the chromatin interactions involving the promoters of four types of genes annotated by GENCODE 19, namely, lincRNAs, antisense ncRNAs, microRNAs and protein-coding genes. The network properties indicated that these ChINs were scale-free like^21^ with power-law exponents (Supplementary Fig. S1B, and the basic network descriptors are shown in Supplementary Fig. S1D). The ChIN of the K562 cells contained 1309 components (or disconnected sub-networks), and the largest is shown in Fig. 1A and contains many known lincRNA genes. In total, 692 (approximately 9.7%) lincRNA genes were involved in the ChIN, of which 46% had expression levels of more than 0.1 RPKM. Another 24% had expression levels of less than 0.1 RPKM, and the remaining 30% had expression levels of 0 RPKM. Comparatively, the genes that were involved in the ChIN included 44.4% of the known protein-coding genes, 30.8% of the antisense genes, and 14.9% of the miRNAs. When the genes involved in the ChINs of the K562 and MCF7 cells were compared, a smaller proportion of ncRNA genes overlapped between the K562 and MCF7 cell lines, while a larger proportion of protein-coding genes overlapped (Fig. 1B, 59% for K562 and 83.7% for MCF7). This indicates that the ncRNA genes were more cell-specific in the ChINs. The expression levels of the lincRNA genes in the ChIN were higher than those not in the ChIN (p-value < 2.2E-16, Wilcoxon rank-sum test) (Supplementary Fig. S1C and Supplementary File 2). A comparison of degree distributions showed that the lincRNA genes in the ChIN had the smallest degrees on average (Fig. 1C), while the protein-coding genes had the largest degrees. These results suggest that the whole chromatin interaction network was generally shaped around protein-coding genes, but not ncRNA genes. The analysis showed that the lincRNA genes were involved in the chromatin interaction network, but they may not generally be the hubs of the network.

Based on chromatin interactions and RNAPII binding signals, genes can be classified into three different transcription models with distinct genomic properties^17^: basal promoter model, single-gene model, and multi-gene model. In addition, we further divided the lincRNA genes in the multi-gene model into two categories (see the Methods section): Category 1 (C1): “interacting with protein-coding genes” (Fig. 1D, for example TERC); and Category 2 (C2): “interacting with genes other than protein-coding genes, such as other lincRNAs or anti-sense non-coding RNAs” (Fig. 1E, for example RP11-671C19.1). To obtain a comprehensive view of the lincRNA genes through long-range chromatin interactions, we assigned the other lincRNA genes to three other categories: C3 - “single gene model” (Fig. 1F, for example AC073236.3); C4 - “basal promoters”; and C5 - “not transcribed (no chromatin interaction and not transcribed)”. Statistical analysis of the lincRNA genes belonging to these five categories (Fig. 1G) showed that the majority of the lincRNA genes (81.7% for K562 cells and 84.7% for MCF7 cells) were not involved in either chromatin interactions or RNAPII binding, indicating that most annotated lincRNAs are cell specific and not transcribed in the K562 and MCF7 cell lines. Regarding the lincRNA genes that exhibited either chromatin interactions or RNAPII binding, the lincRNA genes in C1 were transcribed more actively (Fig. 1H). Another interesting result was that most (>86%) lincRNA promoters in the ChINs belonged to C1 (Fig. 1G) with promoter-promoter interactions, suggesting that the lincRNA and protein-coding genes may be organized into a larger co-transcription framework.

Previous studies^17^,^21^ have shown that interacting genes tend to share the same “transcription factory” and possess combinatorial regulatory functions. To elucidate whether the “multi-gene” complexes were organized into functional compartments^21^, we sorted the ChINs into multiple communities using the ModuLand method^23^. The ChINs in the K562 and MCF7 cells consisted of 1513 and 1550 communities, respectively. Among the communities that had twenty or more nodes, 67.2% (82/122 from the K562 cells) and 68.9% (31/45 from the MCF7 cells) contained lincRNA genes, suggesting that the lincRNA genes were widely distributed in the ChINs. All of the communities were enriched in multiple functions, and these functions were distinct among the communities and cell lines. We observed at least 20 gene ontology (GO) terms in each of the qualified 122 communities in the K562 cells, and 44.6% of the observed GO terms only appeared in one community, suggesting that the ChINs were organized into functional components. Similar observations were also made in the MCF7 cells.

### Transcription regulation of lincRNA genes with distal regulatory elements (DREs)

The transcription of lincRNAs can be regulated by distal regulatory elements (DREs), which are defined as genomic regions that do not overlap with any promoter regions of known genes in the GENCODE annotation. Based on the current understanding, DREs can be brought proximal to the promoters of their target genes through DNA looping to regulate the expression of their target genes. More than 97% of the interactions between lincRNA genes and DREs were on the same chromosome, and the genomic distances were mostly less than 1 Mb (Supplementary Fig. S2A,B). LincRNA genes tended to link one DRE, although there were a few exceptions with as many as 126 DREs (Supplementary Fig. S2C).

**Figure 2:**
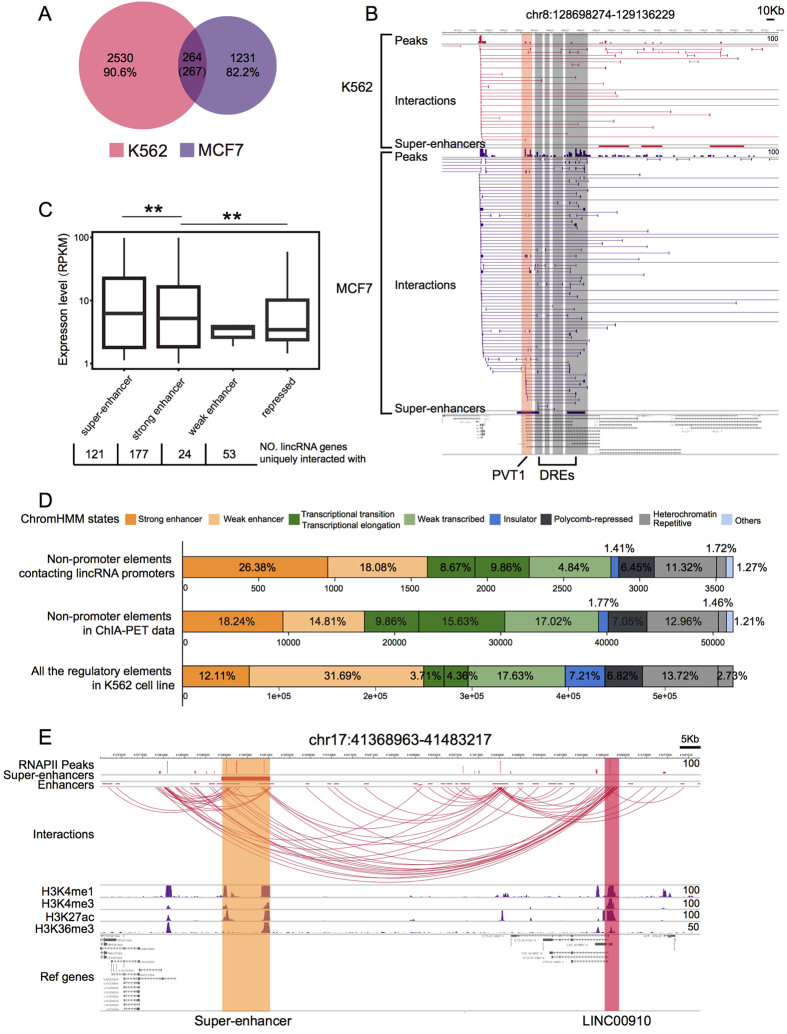
Transcription regulation of lincRNAs with distal regulatory elements (DREs). (**A**) A Venn diagram of DREs interacting with lincRNA promoters in K562 and MCF7 cells. Compared to Fig. 1B, the smaller proportion of common DREs between the K562 and MCF7 cells shows that the DREs are more cell-specific. (**B**) An example of a MCF7-specific lincRNA PVT1 and its interactions with DREs. (**C**) The number of lincRNA genes exclusively interacting with super-enhancers, strong enhancers, weak enhancers, and repressed regions, as well as their expression levels (RPKM) in K562 cells. (**D**) Chromatin states of DREs defined using ChromHMM in K562 cells. (Upper) DREs interacting with lincRNAs; (Middle) DREs from the ChIA-PET data; (Bottom) DREs from the K562 cell line. The category “others” corresponds to the different types of promoters (strong, weak, or poised) defined by ChromHMM, but not defined as promoter regions by GENCODE gene annotation. (**E**) An example of a super-enhancer regulating lincRNA promoter in K562 cells.

When the lincRNA promoters and their DREs from the chromatin interactions were compared separately, the DREs were more cell-specific than the lincRNA promoters (Fig. 1B for lincRNAs and Fig. 2A for DREs). Of the 1088 lincRNA promoters anchored by ChIA-PET in the K562 cell line, 507(46.6%) were also in the MCF7 cell line. Most of these common lincRNA genes (443, 87.4% of 507) were associated with at least one additional DRE. For example, lincRNA PVT1 was amplified in primary breast tumors^24^, and its expression level was higher in MCF7 cells than in K562 cells (Reads Per Kilo bases per Million reads - RPKM ratio = 2.1). The ChIA-PET data showed that PVT1 was anchored to multiple enhancers within its genebody in the MCF7 cells (including one super-enhancer), but not in the K562 cells (Fig. 2B).

To better understand the functional roles of DREs interacting with lincRNAs, we mapped them to different chromatin states defined by ChromHMM^25^. The results showed that DREs associated with lincRNA genes exhibited higher proportions of strong enhancers and lower proportions of weak enhancers compared with all the regulatory elements in human genome (Fig. 2D for K562 and Supplementary Fig. S2D for MCF7).

DREs with active or repressed chromatin states were differentiated by distinct histone marks (e.g., H3K27ac and H3K4me1 for strong/weak enhancers and H3K27me3 for repressed regions)^25^, which may impact the transcription of their target genes in different ways. We measured the expression levels of the lincRNA genes associated with DREs belonging to strong/weak enhancers and repressed regions. The expression levels of the lincRNA genes regulated by strong enhancers were significantly higher than those regulated by repressed regions (p-value < 0.01, Wilcoxon rank-sum test) (Fig. 2C).

Super-enhancers are groups of enhancers that are proximal to the genes that control cell identity^26^. In cancer cells, super-enhancers are found proximal to genes with known oncogenic functions^27^. Super-enhancers may also influence the transcription of lincRNA genes through long-range chromatin interactions. We overlapped super-enhancers defined on the basis of H3K27ac signals^26^ with ChIA-PET DREs and found that 540 out of 742 super-enhancers in the K562 cells contained at least one ChIA-PET DRE, and more than half of the super-enhancers overlapped with two or more DREs (Supplementary Fig. S2E,F). Permutation tests^28^ showed that the super-enhancers were highly enriched in the areas where they co-localized with DREs (p-value < 0.001). In total, 121 lincRNA promoters interacted with the super-enhancers, and their degrees and expression levels were significantly higher than those that did not interact with the super-enhancers (p-value < 0.01, Wilcoxon rank-sum test) (Fig. 2C and Supplementary Fig. S2G). The lincRNAs that were associated with super-enhancers in the K562 cells, but not the MCF7 cells, showed significantly higher expression levels and vice versa (p-value < 1.606E-6, analyzed by a paired t-test) (Supplementary Fig. S2H,I). For example, LINC00910 was a highly connected gene that interacted with 47 promoter regions in the ChIN and contacted 126 DREs in the K562 cells (Fig. 2E). It linked to an upstream super-enhancer that overlapped with two DREs. Gene set enrichment analysis (GSEA) using 108 sets of RNA-Seq expression data from 55 cell types (see the Methods section) revealed that it was involved in immune-related functions, such as lymphocyte activation and humoral immune response.

### LincRNA loci act as enhancers through chromatin interactions at the DNA level

To further explore the potential cell-specific functions of lincRNAs through chromatin interactions, we turned our attention to lincRNA-mRNA interactions, as the lincRNA genes in category C1 constituted most of the lincRNA genes within the ChINs (Fig. 1G) and their transcription was more active than the lincRNAs in the other categories (Fig. 1H and Supplementary Fig. S1E–G).

**Figure 3:**
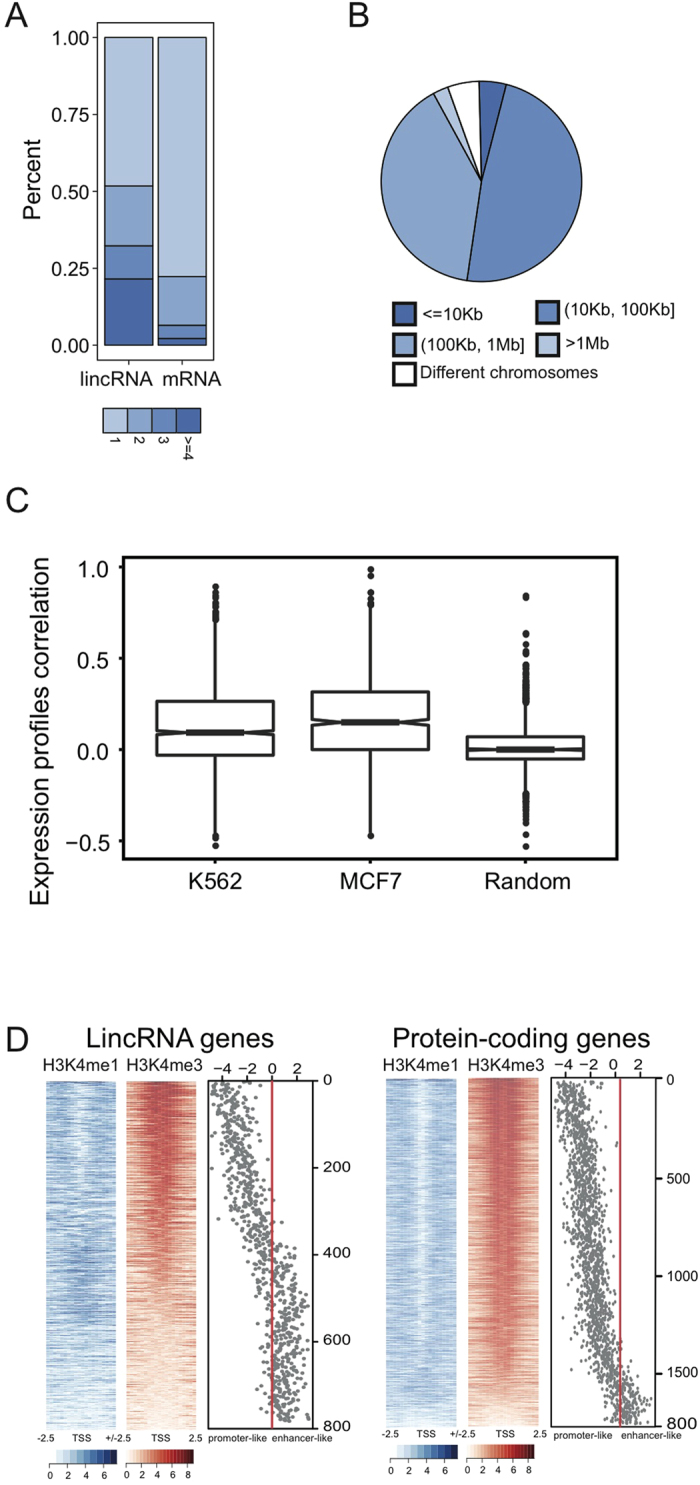
LincRNA loci acting as enhancers at the DNA level through chromatin interactions. (**A**) Degrees of lincRNA and protein-coding genes in the lincRNA-mRNA interaction networks of K562 cells. (**B**) The genomic distance between interacting lincRNA and protein-coding genes in K562 cells. (**C**) Expression correlations between interacting lincRNA and protein-coding genes compared with random gene pairs. (**D**) H3K4me1 and H3K4me3 read coverage, as well as the log2(H3K4me1/H3K4me3), around the TSSs of lincRNA and protein-coding genes in K562 cells.

The lincRNA-mRNA interactions were more cell-specific than the interactions between the protein-coding genes (p-value < 0.033 for K562 cells and p-value < 1.977E-14 for MCF7 cells, as analyzed using the Fisher Exact Test) (Supplementary Table S5). In K562 cells, 2357 such lincRNA-mRNA interactions formed a promoter-promoter interaction network (Supplementary Fig. S3) in which the lincRNA genes were more centralized than the protein-coding genes (Fig. 3A and Supplementary Fig. S4A,B). This result was consistent with the results from other tested cell lines (see Supplementary Fig. S3 for an example in the mESC cell line). Over 50% of the lincRNA genes interacted with two or more protein-coding genes, while only approximately 25% of the protein-coding genes interacted with two or more of the lincRNA genes. When we examined the genomic distance between the interacting genes, the majority of the interacting pairs (94.9%) involved long-range interactions on the same chromosome, with a median distance of approximately 100 Kbs (Fig. 3B). Previous studies^29^,^30^ have found that some lincRNAs (SNAI1, LINC00568, and LINC00570) activate the expression of their neighboring genes. We found that these ncRNA loci were connected to their target genes through chromatin interactions (Supplementary Fig. S5A–C), suggesting that the spatial organization of lincRNA and protein-coding genes may provide a spatial architecture for the lincRNAs to perform their functions.

Expression level analysis revealed that the highly expressed protein-coding genes tended to interact with the lincRNA genes that were also transcribed at higher levels (Supplementary Fig. S4C), which is consistent with previous results involving mouse Bcells^18^. Expression profiles of the 108 RNA-Seq data sets from 55 cell types (see the Methods section) revealed positive correlations between the interacting lincRNA and the protein-coding genes (Fig. 3C), which suggested co-transcription between some of the interacting lincRNA and protein-coding gene pairs.

Recent studies^17^,^31^ have characterized enhancer-associated promoters genome-wide and proven that they can act as enhancers to augment the transcriptional activities of other promoters. LincRNAs have been reported to be enriched with both enhancer-associated and promoter-associated signals^31^,^32^. In our analysis, the lincRNA promoters contacting protein-coding promoters displayed more enhancer-associated marks (H3K4me1 and H3K27ac)^33^ (Supplementary Fig. S1E and S1G) than the other three categories (C2–C4, defined in Fig. 1G), suggesting that these lincRNA promoters possessed potential enhancer-like chromatin states. We hypothesized that a subset of lincRNA promoters exhibit enhancer-like chromatin states, impacting the transcription of their interacting partners through long-range interactions. Since the ratio of H3K4me1/H3K4me3 is commonly used to distinguish between promoters and enhancers, we sought to calculate the read coverage of both the H3K4me1 and H3K4me3 signals, as well as the ratio of log2(H3K4me1/H3K4me3) over intervals surrounding the transcription start sites (TSSs) of the lincRNAs (C1). We also performed an equivalent analysis of the interacting protein-coding genes as a comparison (see the Methods section).

The protein-coding and lincRNA genes had distinct log2(H3K4me1/H3K4me3) signals. As expected, the protein-coding genes exhibited stronger promoter marks than the lincRNAs, but they did not exhibit stronger enhancer marks (Fig. 3D). In total, 42.6% of the lincRNAs were associated with the dominant H3K4me1 histone mark, compared to only 10.27% of the protein-coding genes with a higher H3K4me1 histone mark. We observed similar results in the MCF7 and mouse ESC cell lines (Supplementary Fig. S6). We divided the promoters of the lincRNAs in the lincRNA-mRNA interactions into enhancer-like and promoter-like groups (see the Methods section) and found that the enhancer-like lincRNAs belonged primarily to the strong/weak enhancer states defined by the ChromHMM (Supplementary Fig. S4D). We compared the lincRNA genes with these two chromatin states and found that some properties differed (Supplementary Fig. S4E–G), including the number of isoforms, the number of neighbors in the ChINs, and the distance to their interacting protein-coding genes. Protein-coding genes interacting with enhancer-like lincRNA promoters showed higher expression levels on average than the other categories, although the differences in expression levels were not statistically significant (p-value < 0.11, Wilcoxon rank-sum test) (Supplementary Fig. S4J). Our analysis showed that the lincRNA promoters interacting with protein-coding genes had two distinct chromatin states. Recent studies have also suggested that enhancers can generate non-coding enhancer RNAs^34^,^35^. Whether their transcripts exhibit different functions and how they regulate the transcription of target genes should be explored further in future studies.

### LincRNAs regulate their target genes at the RNA level based on genome spatial organization

**Figure 4:**
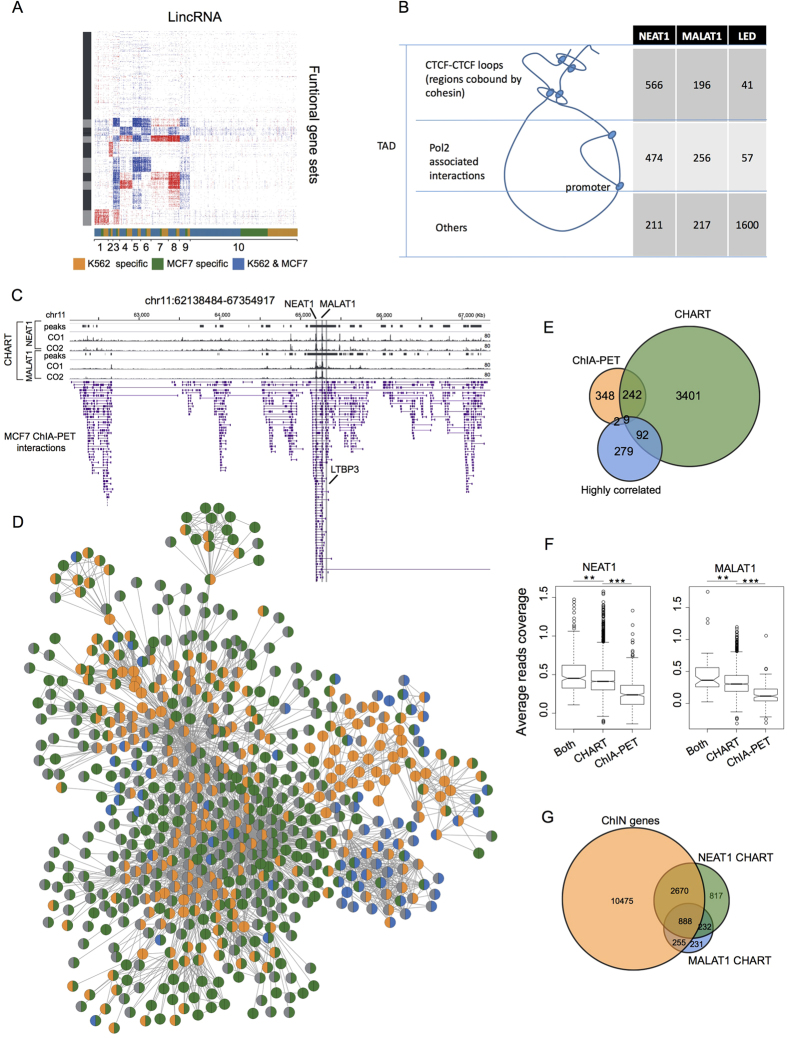
LincRNAs regulating their target genes at the RNA level based on genome spatial organization. (**A**) An expression-based association matrix of lincRNA genes (columns) and functional gene ontology term sets (rows). Red - positive correlation; Blue - negative correlation; White - no correlation. Columns and rows are both clustered using k-means clustering (k = 10). (**B**) Classification of the binding sites of NEAT1, MALAT1 and LED based on chromatin interactions. (**C**) Interacting clusters around the NEAT1 and MALAT1 loci of chromosome 11 in MCF7 cells. The CHART peaks and read coverage of NEAT1 and MALAT1 are also shown. CO1 and CO2 for two different capture oligonucleotides. (**D**) A network of NEAT1- and MALAT1-interacting genes in MCF7 cells (extending to at most three hops). Colors in the left half of the nodes denote those bound by NEAT1 or MALAT1 in CHART; colors in the right half of the nodes denote those interacting with NEAT1 or MALAT1. Blue color denotes those interacting with or bound by NEAT1; green color denotes those interacting with or bound by MALAT1; orange color denotes those interacting with NEAT1 and MALAT1 or bound by NEAT1 and MALAT1. (**E**) A Venn diagram of the genes that NEAT1 interacts with (extending to at most three hops), NEAT1-binding genes and genes whose expression correlates with NEAT1. (**F**) Average read coverage of CHART signals among genes that both interact with and are bound by NEAT1 or MALAT1, or those that only interact with them or are only bound by them. (**G**) Overlap among the genes of the ChIN in MCF7 cells and all of the genes bound by NEAT1 and MALAT1.

LincRNAs lack functional annotations on a large scale. One of the main challenges in the study of lincRNAs involves predicting their functions, either experimentally or computationally. Previous studies^36^,^37^ have used the “guilt-by-association” method to connect lincRNAs to functional gene sets through the high correlation of co-expressed genes. Based on this method, we calculated the correlations of expression profiles between each lincRNA locus and all of the protein-coding genes, and then we performed GSEA^38^ analysis to assign function sets to the lincRNA genes in the ChINs (see the Methods section). This method identified several function-associated clusters of lincRNAs (Fig. 4A and Supplementary Fig. S7A), suggesting that lincRNAs may have diverse functions.

LincRNAs can interact with genomic loci by recruiting proteins^39^ or through direct nucleic acid hybridization^40^. Several technologies, such as capture hybridization analysis of RNA targets (CHART)^41^,^42^ and chromatin isolation by RNA purification (ChIRP)^43^, have been developed to identify the genomic binding sites of endogenous RNAs. CHART-Seq data analysis with NEAT1 and MALAT1 from the MCF7 cell line has revealed that both NEAT1 and MALAT1 prefer to bind to active genomic sites and they co-localize at many regions^41^. Most of their binding regions are inside the gene bodies (Supplementary Fig. S7B). The lncRNA LED prefers to bind at the intergenic regions and is essential for the acetylation of H3K9 at enhancers^44^. According to the ChIA-PET data, NEAT1 and MALAT1 were co-transcribed and highly connected in all of the six examined cell lines (Supplementary Table S6, Supplementary Figs S7C and S8), while LED was part of the C5 (no contact and not transcribed) model.

We intersected the binding sites of NEAT1, MALAT1 and LED with regulatory elements defined by the ChromHMM^45^ and tried to identify the dominant states of their occupancy sites (Supplementary Table S7). We found that the binding sites of NEAT1 and MALAT1 were strongly associated with gene promoters (p-value < 0.001, permutation test^28^), while LED’s binding sites were not.

NEAT1 and MALAT1 targeted the CTCF binding sites and active promoters, which was consistent with the fact that they were bound to active elements^41^. Chromosomes are organized into megabase-sized topologically associating domains (TADs) whose boundaries are occupied by CTCF sites and cohesin^15^,^46^. On sub-domain levels, CTCF and cohesin also mediate the constitutive interactions^47^,^48^. RNAPII can mediate transcription-related chromatin interactions between promoters and their regulatory elements. We next attempted to determine whether the binding sites of NEAT1, MALAT1 and LED were involved in long-range chromatin interactions. We used CTCF- and RNAPII-mediated chromatin interaction data and classified the CTCF interactions into TAD/sub-domain levels based on the cohesin ChIP-Seq data. We then classified the binding sites of the lincRNAs into three categories based on their participation in chromatin interactions: TAD/sub-TAD level (involved in CTCF interactions and co-bound by cohesin), transcriptionally involved interactions (involved in RNAPII interactions), and others. The binding sites of NEAT1 and MALAT1 were involved in both the CTCF- and RNAPII-mediated chromatin interactions (Fig. 4B). The above results suggest that the 3D genome organization impacted the binding sites of NEAT1 and MALAT1. At the same time, high proportions of CTCF-associated binding sites may reflect the roles of lincRNAs in mediating long-range chromatin interactions. The binding sites of LED were not involved in the CTCF- or RNAPII-mediated chromatin interactions. However, we found that LED preferred enhancers to promoters (Supplementary Table S7), which was consistent with previous studies showing that LED is a p53-induced lincRNA and acts on enhancers^44^.

We then focused on NEAT1 and MALAT1. Figure 4C shows an interacting cluster formed by NEAT1, MALAT1 and nearby genes in a region spanning approximately 17.9 Mb, in which MALAT1 interacts with its target gene LTPB3. MALAT1 is located approximately 60 Kb upstream of the LTBP3 promoter. It directly interacts with transcription factor Sp1 and is recruited to the promoter of LTPB3^49^. ChIA-PET data showed that chromatin loops provided spatial proximity for these two genes. If we extended the interacting clusters from one hop to three hops of connectivity (with two intermediate interacting regions), 627 genes were within an inter-connected cluster with 2641 edges (Fig. 4D). The CHART data^41^ showed that 251 of the 601 NEAT1 interacting genes were also bound by NEAT1 at the RNA level (Fig. 4E). The overlapping portion between the NEAT1-binding genes and the NEAT1-interacting genes was comparable to the overlapping portion between the NEAT1-binding genes and the NEAT1 highly correlated genes. This suggests that the chromatin spatial organization around the lincRNA loci impacted their genomic binding sites, and these results could be used to predict the lincRNA target genes. Similar results were observed for MALAT1 (Supplementary Fig. S7E). We divided the genes in the interacting cluster into three groups: (1) bound by lincRNAs and interacting with lincRNA genes, (2) only bound by lincRNAs, (3) only interacting with lincRNA genes. The expression correlations between lincRNA and their targets (genes in (1) and (2)) were significantly higher than the other genes in the interacting cluster (p-value < 0.05) (genes in (3)) (Supplementary Fig. S7F), suggesting that some lincRNA binding events were functional. The CHART read coverage pertaining to the genes bound by lincRNAs and interacting with lincRNA genes were significantly higher than those only bound by lincRNAs (p-value < 0.01) (Fig. 4F), suggesting that lincRNA binding sites spatially proximal to lincRNAs have a higher binding affinity than distant binding sites. The genes in categories (1) and (3) were all spatially proximal genes for NEAT1 or MALAT1. The read coverage in (1) was significantly higher than in (3) (p-value < 0.0001), suggesting that only a portion of the proximal genes were bound by lincRNAs (Fig. 4F) and that other factors besides genome organization helped to determine lincRNA binding sites. Across the whole genome, NEAT1 and MALAT1 bound to thousands of genes, and over 60% of their target genes were mapped to the ChINs, which were distributed in hundreds of communities (Supplementary Fig. S7G,H). Because the communities with multiple genes were functional components of the ChINs^21^, this result indicates that a single lincRNA may interact with many genes in different communities and have various functions.

Some of the other lincRNA-binding events showed similar results. Firre has been shown to bind to the genic regions of Slc25a12, Ypel4, Eef1a1, Atf4 and Ppp1r10 in mouse ESCs^9^. ChIA-PET data has shown that these five genes were all within three hops of connectivity of Firre in mESCs (Supplementary Fig. S9A), indicating proximity between Firre and these genes. Nanog, Sox2 and Fgf4^50^, three target genes of the lincRNA TUNA, were also found to be within three hops of connectivity of TUNA in mESCs (Supplementary Fig. S9B).

We hypothesized that, like NEAT1 and MALAT1, other highly connected lincRNA genes in ChINs would also be bound to their interacting genes, and the functions of these interacting genes may be related. We then explored the functions of the neighboring genes of the top ten connected lincRNAs. For NEAT1 and MALAT1, their neighbors had many distinct functions in the K562 and MCF7 cell lines. In K562 cells, many of the neighboring genes of the top connected lincRNAs were enriched in functions associated with genome structures, including nucleosome assembly, DNA packaging, and chromatin organization. In MCF7 cells, some were enriched in pathways involved in ureteric bud formation.

### Cell-line specificity of chromatin interactions for lincRNA genes

Cell-specific genes often show cell-specific expression levels, and cell-specific interactions provide a structural basis for cell-specific transcription^17^,^22^. We compared the expression levels of lincRNAs exclusively with interactions in K562 and MCF7 cells. Of the lincRNA genes with interactions, 679 (51%) and 350 (35%) of the lincRNAs specific to K562 and MCF7 cell lines showed specific expression patterns in their respective cells (Supplementary Fig. S10A,B), suggesting that cell-specific chromatin interactions play a role in the regulation of lincRNA gene transcription.

**Figure 5:**
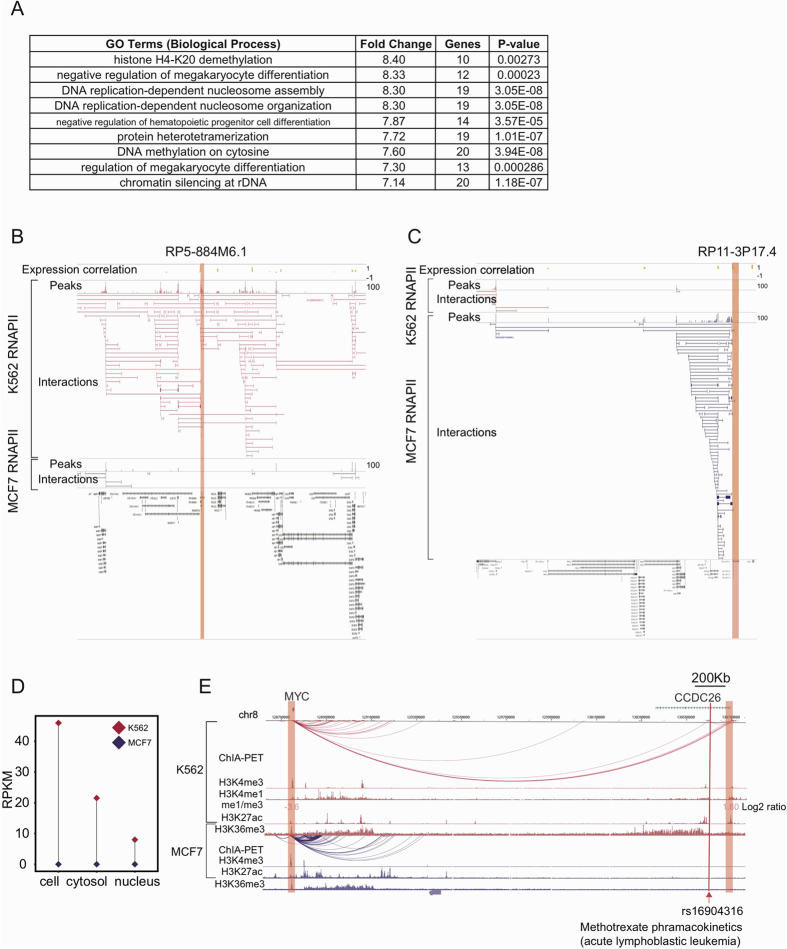
Cell-specific interactions involving lincRNA genes and diseases. (**A**) Functional enrichment of protein-coding genes that interact with lincRNA genes in K562 cells. (**B**) An example of K562-specific interactions involving RP5-884M6.1. (**C**) An example of MCF7-specific interactions involving RP11-3P17.4. (**D**) Expression levels of CCDC26 in K562 and MCF7 cells. (**E**) Interaction between the disease-associated lincRNA locus CCDC26 and MYC.

Of the interactions between the lincRNA and protein-coding genes, 1711 (73.2%) and 572 (47.5%) of the interactions were specific to the K562 and MCF7 cell lines, respectively. We have already shown that the genes involved in the lincRNA-mRNA interactions were co-transcribed (Fig. 3C and Supplementary Fig. S4H,I), indicating that their functions might be related. Consistent with our expectations, a functional enrichment analysis of the protein-coding genes interacting with lincRNAs revealed that the immunity and blood-related functions were enriched in the K562 cells, including the regulation of megakaryocyte differentiation and the regulation of hematopoietic progenitor cell differentiation (Fig. 5A). In the MCF7 cells, the viral life cycle and viral process were enriched, supporting the observation of multiple viruses found to co-exist in human breast cancers^51^ (Supplementary Fig. S10C). The above results demonstrate that chromatin interactions between lincRNA and protein-coding genes are functionally organized and may contribute to cell-specific functions.

We then analyzed the expression profiles of all the annotated lincRNA genes in the 108 RNA-Seq data sets from 55 cell types (see the Methods section) to find genes that were exclusively expressed and also exhibited cell-specific interactions in the K562 and MCF7 ChINs. We identified 21 and 16 lincRNA genes (Supplementary Table S8) in the K562 and MCF7 cell lines, respectively. We conjectured that their functions may depend on the spatial organization around them.

RP5-884M6.1 was exclusively expressed in the K562 cells (Supplementary Fig. S10D). It was located in the human genome region 7q22, a commonly deleted region previously identified in myeloid leukemia^52^. Its neighboring gene PIK3CG was involved in multiple signaling pathways, including leukocyte activation and migration^53^. We observed abundant chromatin interactions with RP5-884M6.1 in the K562 cells, but not in the MCF7 cells (Fig. 5B). Its expression profile correlated well with its interacting genes, suggesting a cell-specific co-transcription mechanism.

RP11-3P17.4 is a MCF7-specific gene (Supplementary Fig. S10E) that interacted with the two protein-coding genes SPTSSB and NMD3, as well as multiple DREs upstream in the MCF7 cells, but not in the K562 cells (Fig. 5C). Intriguingly, some of its DREs were detected with RNAPII peaks, suggesting that RP11-3P17.4 may potentially be regulated by several transcribed enhancers in MCF7.

The above examples suggest that cell-specific chromatin interactions involving lincRNA genes affect cell-specific lincRNA expression profiles.

### SNP-associated chromatin interactions and diseases

LncRNAs are recognized to be involved in many human diseases^2^, including breast cancer^24^,^54^ and leukemia^54^,^55^. Genome-wide association studies (GWASs) have identified numerous diseases or trait-associated single nucleotide polymorphisms (SNPs), and the majority of these SNPs are located in the non-coding portions of the genome. The target genes of these SNPs from non-coding portions are generally unknown, which is one of the main challenges to post-GWAS research. Some studies have already shown that these non-coding regions may influence the expression of genes through long-range chromatin interactions^56^.

GWAS catalogs^57^ are collections of SNPs from published studies. We mapped SNPs from GWAS catalogs to genes through chromatin interactions in the ChINs, with the assumption that the interacting genes were potential target genes of these SNPs. There were 784 and 541 SNPs mapped to genes in the ChINs of the K562 and MCF7 cells, respectively, including 21 lincRNA and 47 antisense genes (Supplementary Table S9). There were 72 SNPs mapped to lincRNA genes involved in lincRNA-mRNA interactions, including 31 enhancer-like lincRNAs. In the K562 cells, several of the SNPs were associated with blood-related traits. For example, CCDC26 is a lincRNA locus located approximately 1.94 Mb upstream of the MYC gene promoter and was expressed exclusively in K562 cells (RPKM > 1) (Fig. 5D), and several studies have suggested that it is related to Acute Myeloid Leukemia (AML)^58^,^59^. GWAS data from children with newly diagnosed acute lymphoblastic leukemia (ALL)^60^ uncovered a SNP (rs16904316) inside the CCDC26 genebody. ChIA-PET data identified long-range interactions between MYC and CCDC26 in K562 cells, but not in MCF7 cells (Fig. 5E). Notably, the CCDC26 promoter regions overlapped with a super-enhancer region, suggesting it has enhancer-like roles. ChIA-PET data can provide evidence for physical connections between disease-related genes and their distal regulatory elements. In these interactions, lincRNA loci may act as both target genes of SNPs and distal regulatory elements of other genes. Based on these observations, it’s reasonable to postulate that the interactions of lincRNAs with GWAS-identified SNPs and protein-coding genes may contribute to specific diseases.

## Discussion

With the progress of next-generation sequencing, an increasing number of non-coding RNAs have been identified^61^. However, the functions of these non-coding RNAs have not been adequately explored. In this study, we systematically characterized the functions of lincRNAs in transcription regulation through long-range chromatin interactions. By building chromatin interaction networks (ChINs) consisting of both lincRNA and protein-coding genes, we found that, although a small proportion of the known lincRNA genes participated in chromatin interactions, they were widely distributed in the functionally organized ChINs. These results suggest that lincRNAs may be involved in different regulatory activities. The expression levels of the interacting lincRNA and protein-coding genes in the 108 RNA-Seq data from 55 cell types were positively correlated on average, suggesting that the lincRNA and protein-coding genes are organized into larger, co-transcriptional networks.

LincRNA genes can be regulated by distal regulatory elements through chromatin interactions, and those regulated by super-enhancers exhibited higher expression levels and had higher interaction degrees. A previous study^62^ has mapped the target partners of lincRNAs to *cis* regulatory elements annotated by the Encyclopedia of DNA Elements (ENCODE) Consortium^63^. Both studies revealed that the expression of lincRNAs is regulated by different types of distal regulatory elements, which is similar to the regulation of protein-coding genes with distal regulatory elements. However, the combinatorial patterns of the chromatin states and the transcription factor binding sites in the promoter regions and distal regulatory elements are distinct between the lincRNAs and protein-coding genes^64^.

Similar to microRNAs, most of the lincRNA genes within the ChINs interacted with protein-coding genes^22^. More than half of the lincRNA genes interacted with two or more protein-coding genes, while less than 25% of the protein-coding genes were in contact with two or more lincRNA genes. This suggests that a single lincRNA locus was proximal to multiple protein-coding genes during transcription. A substantial subset of lincRNA promoters were associated with enhancer-like chromatin states (higher H3K4me1 signals than H3K4me3 signals), which has been reported in previous studies^17^,^31^. These results suggest potential regulatory properties of these lincRNA promoters, although the exact mechanisms need to be further elucidated. Our study also confirms the results from previous studies demonstrating that enhancers are widely transcribed in the genome^34^,^35^.

One of the main objectives of the study of lincRNA is to identify the target genes that they transcriptionally regulate. Different strategies have been proposed for this purpose: (1) searching the nearby sense or anti-sense genes of the lincRNAs; (2) examining the co-expression of lincRNAs and other genes; (3) isolating the target genes of a specific lincRNA with ChIRP/CHART; or (4) conducting loss-of-function or gain-of-function experiments involving a specific lincRNA^65^. LincRNAs have been shown to regulate the transcription of other genes and modify the chromatin states through either RNA-DNA or RNA-protein-DNA interactions^66^. Previous studies^41^,^44^ have identified hundreds of binding sites for lincRNAs using technologies such as CHART^41^ and ChIRP^44^. In this study, we proposed to identify the target genes of lincRNAs through chromatin interactions. Our hypothesis was that the transcribed lincRNAs would perform their functions in the local space, which would save energy and make it easier to find their target genes. By comparing interaction anchors from the ChIA-PET data and binding sites from the CHART/ChIRP MCF7 cell line, we found that the binding sites of NEAT1 and MALAT1 were associated with NEAT1/MALAT1 interaction anchors from transcription-related (RNAPII) or constitutive (CTCF) chromatin interactions at a maximum of three hops (p-value < 0.001, as analyzed by the Permutation Test, z-score = 30.257 for NEAT1, and z-score = 20.351 for MALAT1). Furthermore, the lincRNA binding genes that were proximal to the lincRNA loci (within three hops) had higher CHART signals than those that were spatially distant. These results suggest the possibility that chromatin interactions provide spatial architecture for lincRNAs that are searching for target genes far away in the linear genome or on different, but proximal, chromosomes. Furthermore, 5.77% of the binding sites of LED overlapped with chromatin interaction anchors, although the overlapping portion exceeded what was expected based on the permutation test (p-value < 0.001)^28^. These results suggest that NEAT1/MALAT1 and LED have played different roles in genome organization. It should also be noted that the lincRNAs did not bind to all of the genes in spatial proximity, which implies that factors other than genome organization impact the lincRNA binding profile.

The functional enrichment of protein-coding genes in contact with lincRNA genes of different cell lines was associated with cell-specific diseases. This result revealed the functional roles of the lincRNA-mRNA co-transcriptional network. The cell-specific interactions involving lincRNA genes impacted their expression levels, suggesting that the chromatin interactions, together with protein-coding genes, also provide an architectural context for lincRNA transcription. Disease-associated SNPs within the lincRNA loci may influence their functions and cause diseases. For example, the CCDC26 data showed that enhancer-like lincRNA genes with disease-associated SNPs may regulate oncogenes through long-range chromatin interactions.

Our work suggests that lincRNAs may function at both the DNA and RNA levels. The association of GWAS-identified SNPs with lincRNAs shows that some lincRNAs are disease-associated, which may provide potential candidates for drug targets.

## Materials and Methods

### Data sources

The ChIA-PET data used in this study included the following: the human K562 and MCF7 cell lines, mouse embryonic stem cells (ESCs), mouse neural stem cells (NSCs), mouse neural progenitor cells (NPCs), and mouse B cells. The ChIA-PET data from the two human cell lines (K562, MCF7) were downloaded from ENCODE (https://www.encodeproject.org/), and the data from the four mouse cell lines (ESC, NSC, NPC and Bcell) were downloaded from the SRA archive^67^. The RNA-Seq data and ChIP-Seq data were also downloaded from ENCODE. The lincRNA binding peaks from the CHART and ChIRP data were extracted from relevant publications^41^,^43^. Detailed data sources are listed in Supplementary Table S1.

### ChIA-PET, ChIP-Seq and RNA-Seq data analysis

The raw sequences of the ChIA-PET data were re-processed with the updated ChIA-PET Tool Pipeline^68^. Replicate data sets were merged before processing. Statistics regarding the chromatin interaction clusters of the ChIA-PET data in the two human and four mouse cell lines are shown in Supplementary Table S2.

For the K562 and mouse ESC lines, mapped files (in bigwig format) were downloaded from ENCODE. For the MCF7 cell line, raw data were filtered with adapters and low quality reads were trimmed using Trimmomatic^69^. The clean reads were mapped to the human hg19 genome using bwa^70^, and the BAM format files were converted to Bedgraph format and normalized based on sequencing depth. The read coverage around the TSSs was calculated using Bedtools^71^.

ChIP-Seq and RNA-Seq data were processed with a uniform pipeline. Of the 108 RNA-Seq data sets used in our study, 64 were obtained with the Illumina G2Ax platform and 44 were obtained with the Illumina HiSeq 2000 platform. Since the RNA-Seq data were sequenced using different sequencing platforms, we checked to see if there were any batch effects between the different sequencing platforms. The first two principal components of the expression matrix showed that there were indeed batch effects from the different sequencing platforms (Supplementary Fig. 1A). We then used the combat function in R package sva^72^ to correct for the batch effects. The following analysis was performed on the corrected expression data.

### Genome-wide chromatin interaction network construction

Chromatin interaction anchor regions from the ChIA-PET data were used as raw anchors, and the overlapping, neighboring anchors were merged into larger regions that were treated as nodes in the chromatin interaction network. If two regions (nodes in the network) had chromatin interactions, an edge was added between these two regions (nodes) in the network. Using this method, we constructed the genome-wide chromatin interaction network.

We used the GENCODE^61^ V19 and M4 annotation files to define the promoter regions for human and mouse samples, respectively. Genomic regions that were 2.5 kb upstream and downstream from the TSSs of the annotated genes were considered to be promoter regions. The genomic regions (nodes in the chromatin interaction network) were considered to be interacting promoter regions if they overlapped by at least 1 bp with the promoter regions from the GENCODE gene annotation. Network graphs were saved as additional files in GraphMl format (Supplementary Files 3 and 4). The remaining genomic regions (nodes in the chromatin interaction network) were considered to be DREs.

### Gene types in the chromatin interaction network

We divided the promoters of the genes in the ChINs into the following categories: “protein_coding,” “lincRNA,” “antisense,” “miRNA,” “sense_overlapping,” “sense_intronic” and “processed_transcript”. LncRNA loci that overlapped with any protein-coding genes were classified as “others.”

### Basic interaction network analysis

We calculated several network descriptors of the chromatin interaction network, including scale-freeness (a network with a degree distribution following a power-law distribution), degree distribution (the degree of a node refers to the number of neighbors it has, and the degree distribution is the probability distribution of these degrees over the network), K-core distribution (see the main text), modularity (in our study, we used the ModuLand algorithm^23^ to identify communities representing the modularity of the network), betweenness (the number of shortest paths passing through a node), closeness (the number of shortest paths required to reach any other node in the network), transitivity (clustering coefficient), graph density (the ratio of the number of edges and the number of possible edges), etc. A log-log plot of the network descriptors of the K562 cells is illustrated in Supplementary Fig. 1C. These parameters were calculated using the R platform of the igraph package (www.igraph.org).

### Mapping of ChromHMM states to non-promoter elements

DREs overlapped with the coordinates of each ChromHMM state. Multiple mappings of ChromHMM chromatin states to DREs were ordered based on the following priorities: strong enhancer, weak enhancer, transcriptional transition/elongation, weak transcribed, insulator, polycomb-repressed and heterochromatin/repetitive. DREs that did not overlap with any non-promoter ChromHMM states were classified as others, which were different types of promoter regions than those defined by ChromHMM, but not included in the GENCODE gene promoter region list.

### Classification of lincRNAs into different categories based on chromatin interactions and RNAPII binding

We divided the lincRNA genes of the multi-gene complexes of the ChINs into two categories: C1, interacting with protein-coding genes; and C2, not interacting with protein-coding genes but contacting other genes in the ChINs, including antisense and miRNA genes. We assigned the remaining lincRNAs to three other categories for comparison: C3 - “single gene model (just contacting with DREs)”; C4 - “basal promoters (no chromatin interactions but with RNAPII peaks in the promoter regions)”; and C5 - “not transcribed (no chromatin interaction and not transcribed).” This classification was consistent with the previous study^17^.

### Gene set enrichment analysis (GSEA) of lincRNA genes

Similar to the analyses performed by Pauli *et al*. and Guttman *et al*.^36^,^37^, the expression levels of each lincRNA gene were correlated with the expression levels of the protein-coding genes in the 108 RNA-Seq data sets from 55 cell types from the ENCODE genomic annotation (https://www.encodeproject.org/data/annotations/). The resulting correlation-based rank list of protein-coding genes for each lincRNA gene was then subjected to GSEA to identify the associated GO terms using false-discovery rate of 0.01. An association matrix between lincRNAs (rows) and GO terms (columns) was constructed and clustered using k-means clustering for both rows and columns.

### Community division and functional enrichment

We divided the ChINs into sub components (communities) using the Moduland^23^ algorithm. We performed Gene Ontology (GO) analysis using GOstats^73^ on the communities with at least 20 nodes, and the results were also verified using PANTHER^74^. We then removed the interactions between the lincRNA and protein-coding genes and conducted GO enrichment analysis for protein-coding genes.

### Division between enhancer-like and promoter-like lincRNA promoter regions in the lincRNA-mRNA gene interaction pairs

We used log2(H3K4me1/H3K4me3) to define the chromatin states of the promoter regions. The promoter regions (+/− 2.5 Kb of TSSs) were divided into 20 bins, and the average read coverage of both the H3K4me1 and H3K4me3 signals in each bin was calculated using Bedtools with the option-map^71^ as H3K4me1_20bins_ and H3K4me3_20bins_ and normalized to the sequencing depth. We also calculated the average read coverage in the +/− 1 Kb regions (H3K4me1_2Kb_ and H3K4me3_2Kb_) around the TSSs and sorted H3K4me3_2Kb_ by number. Heatmaps of H3K4me1_20bins_ and H3K4me3_20bins_ marks around the promoter regions were drawn using heatmap.2 in R and ordered by H3K4me3_2Kb_. Log2(H3K4me1_2Kb_/H3K4me3_2Kb_) was used to divide the promoter regions into promoter-like(<0) and enhancer-like(>0) groups. We then divided the interactions between lincRNA and protein-coding genes into four categories (Supplementary Table S4).

### Comparisons between ChIA-PET data and ChIRP/CHART data

ChIRP or CHART data (peaks) pertaining to NEAT1, MALAT1, TERC and HOTAIR lncRNA were downloaded from the relevant publications^41^,^43^ to find the genic-binding regions of these lncRNAs. We defined the genic regions as +/− 2.5 Kb of the genebody, and an overlap of at least 1 bp between the genic regions and RNA-binding regions was counted as the genic binding region of that lncRNA. For the ChIA-PET data, the promoter regions of the genes interacting with a lncRNA promoter were considered to be the interacting regions of that lncRNA gene.

### LincRNAs with cell-specific expression profiles

The normalized RPKM values of the 108 RNA-Seq data sets from 55 human cell types were downloaded from the ENCODE genomic annotation (https://www.encodeproject.org/data/annotations/), and the expression levels of each gene from the different replicates were averaged. To identify the lincRNA genes expressed exclusively in the K562 or MCF7 cells, we calculated the maximum expression levels of all of the subcellular components available (including the cell, chromatin, nucleus, nucleolus, nucleoplasm and cytosol) in the K562 or MCF7 cells, as well as the maximum expression levels of the remaining cell lines or tissues. We then calculated the fold change between the two values. We considered a fold change >= 2 to be an indication that it was exclusively expressed in that cell line. To choose genes that correlated highly with NEAT1 and MALAT1, we plotted correlation distributions among all of the genes in GENCODE V19 (Supplementary Fig. S7D). We found that there was a dip between 0.6 and 0.7 (as shown in Supplementary Fig. S7D), which indicates that the expression correlation between most gene pairs is below 0.6. Therefore, we used 0.6 as the threshold for highly correlated genes.

### Disease-associated SNPs

Disease-associated SNPs were downloaded from the NHGRI GWAS Catalog^57^ (accessed in July of 2015). All of the SNPs were mapped to the lincRNA and protein-coding extended (+/− 2.5 Kb) gene bodies in the ChINs, as well as all of the DREs that interacted with lincRNA promoters.

### Visualizations

The WashU Epigenome Browser^75^ and Integrative Genomics Viewer (IGV)^76^ were used to visualize the long-range chromatin interactions. Customized R scripts were also used to generate the figures in this manuscript.

### P-value Note

NS: Not Significant; *P ≤ 0.05; **P ≤ 0.01; ***P < 0.0001. The Wilcoxon rank-sum test was used to test whether two populations were significantly different if there is no specific illustration. R package regionR^28^ was used to test the colocalization of two genomic regions using a permutation test.

## Additional Information

**How to cite this article**: Cai, L. *et al*. A Comprehensive Characterization of the Function of LincRNAs in Transcriptional Regulation Through Long-Range Chromatin Interactions. *Sci. Rep.* **6**, 36572; doi: 10.1038/srep36572 (2016).

**Publisher’s note:** Springer Nature remains neutral with regard to jurisdictional claims in published maps and institutional affiliations.

## Supplementary Material

Supplementary Information

Supplementary Information

Supplementary Information

Supplementary Information
