# Supplementary material for: A Comprehensive Characterization of the Function of LincRNAs in Transcriptional Regulation Through Long-Range Chromatin Interactions
Source: Sci Rep. 2016 Nov 8;6:36572. doi: 10.1038/srep36572 (PMC5099911; doi:10.1038/srep36572)
Supplement: Supplementary Information [file srep36572-s1.pdf]

Supplemental materials for

Comprehensive Characterization of LincRNAs' functions in transcription regulation through Long-Range Chromatin Interactions

Liuyang Cai, Huidan Chang, Yaping Fang, Guoliang Li*

National Key Laboratory of Crop Genetic Improvement, Agricultural Bioinformatics Key Laboratory of Hubei Province, College of Informatics, Huazhong Agricultural University, Wuhan, Hubei 430070, China.

*Corresponding author

Email addresses:

Liuyang Cai	liuyang_cai@foxmail.com
Huidan Chang	changhuidan@webmail.hzau.edu.cn
Yaping Fang	ypfang@mail.hzau.edu.cn
Guoliang Li	guoliang.li@mail.hzau.edu.cn

Supplementary Figures Legends

Figure S1. Properties of Chromatin Interaction Networks (ChINs) involving non-coding RNAs and protein-coding genes in K562.

A. The first two PCs of expression matrix in 108 RNA-Seq data from 55 cell types of GENCODE V19 genes with color representing different sequencing platforms.

B. Degree distribution of genes in the ChIN.

C. Boxplot of expression levels of lincRNA genes in ChINs compared with those that are not in the ChINs in K562.

D. Log-log plots of network descriptors in K562 ChIN, including degree, betweenness, closeness, transitivity. Below are the modularity and graph density.

E-G. H3K4me1, H3K4me3 and H3K27ac signal distributions of lincRNA genes in different interaction categories.

Figure S2. Transcription regulation of lincRNAs with distal regulatory elements (DREs).

A. Proportion of lincRNA gene and DRE interactions on the same or on different chromosomes in K562.

B. Genomic distances between lincRNA and DREs on the same chromosome in K562.

C. Number of DREs per lincRNA promoter interacted with in K562 and MCF7.

D. Chromatin states of DREs in MCF7. (Up) DREs interacting with lincRNAs; (Middle) DREs in ChIA-PET data; (Bottom) DREs in MCF7 cell line. Category "others" corresponds to different kinds of promoters (strong, weak or poised) defined by chromHMM, but not defined as promoter regions by GENCODE gene annotation. We referred to the paper with PMID 22373907[1] for chromatin states from K562 cell line. We referred to the paper with PMID 24916973[2] for chromatin states from MCF7 cell line. Chromatin state "CTCF" in MCF7 corresponds to chromatin state "insulator" in K562, and chromatin state "CTCF+enhancer" in MCF7 corresponds to chromatin state "weak/poised enhancer" in K562.

E. Overlaps between super-enhancers and ChIA-PET DREs in K562.

F. Number of DREs per super-enhancer overlap with in K562.

G. Degree distribution of lincRNA genes interacted with and not interacted with super-enhancers in K562.

H. Expression levels of lincRNAs with super-enhancers cell-specific in K562, but not in MCF7. Their expression levels are significantly higher in K562 than in MCF7 (p-value < 1.606e-6 from paired t-test for log2(RPKM+1)).

I. Expression levels of lincRNAs with super-enhancers in cell-specific MCF7, but not in K562. Their expression levels are significantly higher in MCF7 than in K562 (p-value < 1.266e-2 from paired t-test for log2(RPKM+1)).

Figure S3. Force-directed layout of lincRNA and protein-coding gene interactions network in K562 and mESC cell lines. (Red) LincRNA genes; (Grey) Protein-coding genes.

Figure S4. LincRNA loci in DNA level acting as enhancers through chromatin interactions.

A. Degree distributions of nodes in the chromatin interaction networks consisting of lincRNA and protein-coding genes interactions in MCF7.

B. Boxplot of degrees of lincRNA and protein-coding genes in lincRNA-mRNA interaction network in K562.

C. Interactions between lincRNA and protein-coding genes from different expression groups in K562.

D. Number of enhancer-like lincRNA genes with different chromatin states in K562.

E. Number of enhancer-like and promoter-like lincRNA genes with different number of isoforms in K562.

F. Distance distribution between protein-coding and lincRNA genes of G2 and G3 in Figure S4H, distance between protein-coding genes and their DREs were taken as a comparison.

G. Boxplot of degree distributions of enhancer-like and promoter-like lincRNA genes in K562.

H. Contour plots of density of interactions between lincRNA and protein-coding genes grouped by log2(H3K4me1/H3K4me3) signals of each gene in K562.

I. Contour plots of density of interactions among protein-coding genes grouped by log2(H3K4me1/H3K4me3) signals of each gene in K562.

J. Expression levels of protein-coding genes associated with enhancer-like, promoter-like and both kinds of lincRNA genes, compared with those contact other genes and only DREs in K562.

Figure S5. Examples of chromatin interactions between activating lincRNA (lincRNA-a) loci and their target genes. (A) TRERNA1 and SNAI1; (B) LINC00568 and ECM1; (C) LINC00570 and ROCK2.

Figure S6. H3K4me1 and H3K4me3 reads coverage as well as the log2(H3K4me1/H3K4me3) around TSSs of lincRNA and protein-coding genes in MCF7 and mESC cell lines.

Figure S7. LincRNAs in RNA level regulating their target genes based on genome spatial organization.

A. GO terms with the most number of lincRNA genes in each cluster in Figure 4A.

B. Overlap between lncRNA chromatin binding regions and annotated gene bodies for NEAT1, MALAT1, TERC and HOTAIR.

C. ChIA-PET interactions, CHART peaks and reads coverage of NEAT1 and MALAT1 on the entire chromosome 11 in MCF7.

D. Expression correlation distribution among all the gene pairs in GENCODE V19.

E. Venn diagram of genes MALAT1 interacts with (extend to at most three hops), MALAT1 binding genes and highly-correlated genes of MALAT1.

F. Expression correlation of genes that both interact with and are bound by NEAT1 or

MALAT1, only interact with them and are only bound by them.
G. Distribution of communities of NEAT1 and MALAT1 target genes.
H. Graph illustrating the relations between DNA-DNA interactions and RNA-DNA interactions.

Figure S8. Interacting cluster of NEAT1 and MALAT1 on the same chromosome in mouse ESC, NSC, NPC and Bcell.

Figure S9. Interacting partners of Firre (A) and TUNA (B) extended to three hops of connectivity in mouse ESC cell line.

Figure S10. Cell-specific interactions involving lincRNA genes.
A - B. Percentage difference of expression levels of cell-specific lincRNA genes with chromatin interactions in K562 and MCF7. (A) using longPolyA RNA-Seq data and (B) using longNonPolyA RNA-Seq data. LincRNAs were divided into three categories: only with chromatin interactions in K562, only with chromatin interactions in MCF7 and with chromatin interactions in both cell lines. “Percentage difference” was calculated as (RPKM in K562- RPKM in MCF7)/RPKM in K562.
C. Functional enrichment of protein-coding genes contacting lincRNA genes in MCF7.
D - E. Expression levels of RP5-884M6.1 and RP11-3P17.4 in K562 and MCF7.

1. Ernst, J. & Kellis, M. ChromHMM: automating chromatin-state discovery and characterization. *Nat. Methods* **9,** 215–216 (2012).
2. Taberlay, P. C., Statham, A. L., Kelly, T. K., Clark, S. J. & Jones, P. A. Reconfiguration of nucleosome-depleted regions at distal regulatory elements accompanies DNA methylation of enhancers and insulators in cancer. *Genome Res.* **24,** 1421–1432 (2014).

Supplementary Tables

Table S1. Data used in this article

**ChIP-seq data**		
K562	RNAPII	wgEncodeHaibTfbsK562Pol2V0416101RawRep1.bigWig
	H3K4me1	wgEncodeBroadHistoneK562H3k4me1StdSig.bigWig
	H3K4me3	wgEncodeBroadHistoneK562H3k4me3StdSig.bigWig
	H3K27ac	wgEncodeBroadHistoneK562H3k27acStdSig.bigWig
MCF7	RNAPII	wgEncodeOpenChromChipMcf7Pol2Sig.bigWig
	H3K4me1	SRR1282220
	H3K4me3	wgEncodeUwHistoneMcf7H3k04me3StdRawRep2.bigWig
	H3K27ac	wgEncodeSydhHistoneMcf7H3k27acUcdSig.bigWig
mouse ESC	H3K4me1	GSM1000121
	H3K4me3	GSM1000124
	H3K27ac	GSM1000126
**Transcription factor binding**	http://hgdownload-test.cse.ucsc.edu/goldenPath/hg19/encodeDCC/wgEncodeRegTfbsClustered/	
**ChIA-PET data**		
K562 MCF7	GSE33664	
mouse ESC NSC NPC	GSE44067	
mouse Bcell	SRP029721	
**Annotation file**		
human	http://www.gencodegenes.org/releases/19.html	
mouse	http://www.gencodegenes.org/mouse_releases/4.html	
**phastCons score**	http://hgdownload-test.cse.ucsc.edu/goldenPath/hg19/phastCons46way/vertebrate/	
**Expression levels**	https://www.encodeproject.	

**in 108 RNA-Seq data from 55 cell types**	org/data/annotations/	
**chromHMM**		
K562	http://hgdownload.cse.ucsc.edu/goldenPath/hg18/encodeDCC/wgEncodeBroadHmm/	
MCF7	http://www.ncbi.nlm.nih.gov/geo/query/acc.cgi?acc=GSE57498	
**Disease-associated SNPs**		
https://www.genome.gov/26525384		

Table S2. Statistics of chromatin interaction clusters of ChIA-PET data in two human and four mouse cell lines

		**Frequency**					
**Distance**	**Interaction type**	**K562**	**MCF7**	**mouse ESC**	**mouse NSC**	**mouse NPC**	**mouse Bcell**
< 100Kb	Intra-chromosomal	24128	10851	3239	1395	1022	3554
[100Kb, 1Mb)	Intra-chromosomal	5652	2345	1022	319	303	729
[1Mb, 10Mb)	Intra-chromosomal	111	103	1013	157	21	28
> 10Mb	Intra-chromosomal	42	17	4965	824	15	19
Different chromosomes	Inter-chromosomal	416	311	99709	11999	243	167

Table S3. Color annotations (Nodes colors in Figure 1A)

**Chrom**	**Color**
chr1	
chr2	
chr3	
chr4	
chr5	
chr6	
chr7	
chr8	
chr9	
chr10	
chr11	
chr12	
chr13	
chr14	
chr15	
chr16	
chr17	
chr18	
chr19	
chr20	
chr21	
chr22	

Table S4. Four categories of the interactions between lincRNA and protein-coding genes

LincRNA genes / Protein-coding genes	Promoter-like	Enhancer-like
Promoter-like	G1	G3
Enhancer-like	G2	G4

Table S5. Number of three types of interactions in K562 and MCF7

Interaction types	K562-specific	MCF7-specific	Common
lincRNA-lincRNA	128	74	46
lincRNA-mRNA	1722	581	635
mRNA-mRNA	12228	2843	5010

Table S6. Number of neighbor interacting partners of NEAT1 and MALAT1 in six cell lines (within 3 hops)

Cell line	Organism	Nodes NO.	Edges NO.
K562	Homo sapiens	90	120
MCF7	Homo sapiens	57	74
ESC	Mus musculus	1742	1857
NSC	Mus musculus	529	577
NPC	Mus musculus	14	15
Bcell	Mus musculus	19	32

Table S7. Chromatin states of binding sites of NEAT1, MALAT1 and LED in MCF7 cell line

LncRNA	CTCF+Promoter	promoter	CTCF+Enhancer	Enhancer	Transcribed	CTCF	Others	Total
NEAT1	527	499	0	130	16	30	49	1251
MALAT1	147	249	0	113	46	30	74	669
LED	6	50	2	96	99	85	1360	1698

Table S8. LincRNA genes exclusively with interactions and expressed in K562 or MCF7.

(A). LincRNA genes exclusively expressed in K562 among 108 RNA-Seq data from 55 cell types and within ChINs only in K562

**Gene Symbol**	**Ensembl Name**
RP4-633I8.4	ENSG00000228423
SLC2A1-AS1	ENSG00000227533
RP5-943J3.2	ENSG00000272931
RP5-1061H20.4	ENSG00000177788
AC007405.6	ENSG00000239467
AC069277.2	ENSG00000189229
CTC-338M12.9	ENSG00000248103
RP1-86C11.7	ENSG00000272468
RP11-462G2.1	ENSG00000237643
RP11-497D6.3	ENSG00000227748
RP5-884M6.1	ENSG00000228742
CCDC26	ENSG00000229140
RP11-326C3.13	ENSG00000270030
RP11-587P21.2	ENSG00000257893
RP11-575F12.1	ENSG00000249345
RP11-495K9.9	ENSG00000256258
MIR144	ENSG00000264066
LINC01029	ENSG00000265843
RP11-671C19.2	ENSG00000264334
C20orf203	ENSG00000198547
XXbac-B444P24.14	ENSG00000273139

(B). LincRNA genes exclusively expressed in MCF7 among 108 RNA-Seq data from 55 cell types and within ChINs only in MCF7

**Gene Symbol**	**Ensembl Name**
AC104135.3	ENSG00000204792
AC104135.4	ENSG00000230836
RP11-167H9.6	ENSG00000244541
RP11-3P17.4	ENSG00000240567
CTB-33O18.1	ENSG00000253768
CTA-392E5.1	ENSG00000253154
RP11-629O1.2	ENSG00000261220
RP11-489N22.3	ENSG00000269909
RP11-379F12.4	ENSG00000232638
RP11-379F12.3	ENSG00000243350
RP11-346D6.6	ENSG00000231131
RP11-321G12.1	ENSG00000259459
RP11-178C3.2	ENSG00000267302
RP11-453A12.1	ENSG00000265000
RP11-347D21.3	ENSG00000237423
AL121578.2	ENSG00000259977

Table S9. LincRNA genes containing disease-associated SNPs.

(A). LincRNA genes containing disease-associated SNPs in K562

Chrom	Start	End	Strand	Ensembl Name	Gene symbol	Related Trait
chr1	38512986	38584697	+	ENSG00000224592	RP5-884C9.2	Amyotrophic lateral sclerosis (sporadic);chr1:38528443-38528443;
chr1	47562325	47644943	-	ENSG00000225506	CYP4A22-AS1	Adverse response to chemotherapy (neutropenia/leucopenia) (etoposide);chr1:47632734-47632734;
chr1	182098445	182283196	-	ENSG00000228918	GS1-122H1.2	HDL cholesterol;chr1:182199750-182199750;Cardiovascular heart disease in diabetics;chr1:182112825-182112825;HDL cholesterol;chr1:182199750-182199750;
chr1	227581292	227618736	+	ENSG00000234277	CTD-2090I13.1	Height;chr1:227610249-227610249;
chr1	59250823	59365384	+	ENSG00000234807	RP4-794H19.2	Obesity-related traits;chr1:59296796-59296796;Response to antipsychotic therapy (extrapyramidal side effects);chr1:59333489-59333489;
chr10	28721937	28784742	-	ENSG00000237128	RP11-351M16.3	Preeclampsia;chr10:28738294-28738294;
chr11	86666661	86712001	+	ENSG00000246523	RP11-736K20.6	Adverse response to chemotherapy (neutropenia/leucopenia) (doxorubicin);chr11:86703240-86703240;
chr14	102100791	102197445	-	ENSG00000258404	RP11-1029J19.5	Behavioural disinhibition (generation interaction);chr14:102129288-102129288;
chr14	62182276	62217815	-	ENSG00000258667	HIF1A-AS2	Iron status biomarkers;chr14:62187980-62187980;Attention deficit hyperactivity disorder;chr14:62211585-62211585;
chr14	103011591	103022163	+	ENSG00000259230	CTD-2555C10.3	Large B-cell lymphoma;chr14:103018488-103018488;
chr15	69755365	69863775	+	ENSG00000245750	RP11-279F6.1	Height;chr15:69755818-69755818;
chr15	67695957	67814182	-	ENSG00000259673	IQCH-AS1	Body mass index;chr15:67788548-67788548;Obesity;chr15:67750719-67750719;Obesity;chr15:67750719-67750719;Body mass index;chr15:67788548-67788548;Restless legs syndrome;chr15:67744514-67744514;Body mass index;chr15:67794500-67794500;Restless legs syndrome;chr15:67744514-67744514;
chr15	74781943	74809300	+	ENSG00000261775	RP11-10O17.3	Diastolic blood pressure;chr15:74785026-74785026;Systolic blood pressure;chr15:74785026-74785026;Blood pressure;chr15:74785026-74785026;Diastolic blood pressure;chr15:74785026-74785026;
chr16	58767816	59142878	+	ENSG00000245768	RP11-410D17.2	Blood metabolite ratios;chr16:58795886-58795886;
chr17	3880397	3885193	+	ENSG00000263312	RP11-459C13.1	Smoking behavior;chr17:3883613-3883613;

chr18	72259010	72265744	-	ENSG00000264247	LINC00909	Cervical cancer;chr18:72259522-72259522;
chr19	49834874	49843865	-	ENSG00000197813	CTC-301O7.4	Blood trace element (Se levels);chr19:49834979-49834979;
chr2	65663845	66311773	+	ENSG00000204929	AC074391.1	Age-related hearing impairment (interaction);chr2:66048174-66048174;Bipolar disorder (body mass index interaction);chr2:66031567-66031567;
chr2	9778901	9789568	+	ENSG00000240687	RP11-521D12.1	Self-rated health;chr2:9784258-9784258;
chr20	48909257	48931459	+	ENSG00000203999	RP11-290F20.1	Anger;chr20:48925063-48925063;
chr21	35577356	35697334	+	ENSG00000214955	AP000318.2	Obesity-related traits;chr21:35641323-35641323;
chr3	195869507	195887761	+	ENSG00000224652	LINC00885	Bronchopulmonary dysplasia;chr3:195870036-195870036;
chr3	119813742	119855630	+	ENSG00000242622	RP11-18H7.1	HDL cholesterol;chr3:119841759-119841759;
chr4	14911585	15003669	-	ENSG00000247624	CPEB2-AS1	Political ideology;chr4:14965545-14965545;
chr4	38422283	38524801	-	ENSG00000249534	RP11-83C7.2	Obesity-related traits;chr4:38463850-38463850;
chr6	37475124	37503392	+	ENSG00000204110	RP1-153P14.8	Cognitive performance;chr6:37483920-37483920;
chr6	24742530	24752149	+	ENSG00000224164	RP3-369A17.4	Neutrophil count;chr6:24749185-24749185;
chr6	14977019	15090234	-	ENSG00000234261	RP11-146I2.1	Multiple sclerosis (severity);chr6:15088920-15088920;
chr6	4136306	4157619	+	ENSG00000234817	RP3-400B16.1	Blood metabolite levels;chr6:4145964-4145964;
chr6	30201816	30293911	-	ENSG00000270604	HCG17	Anger;chr6:30211645-30211645;QT interval in Tripanosoma cruzi seropositivity;chr6:30256461-30256461;Autism spectrum disorder, attention deficit-hyperactivity disorder, bipolar disorder, major depressive disorder, and schizophrenia (combined);chr6:30206354-30206354;Schizophrenia;chr6:30206354-30206354;
chr6	25992890	26002003	+	ENSG00000272462	U91328.19	Iron status biomarkers;chr6:25997230-25997230;
chr8	130363937	130692485	-	ENSG00000229140	CCDC26	Methotrexate pharamacokinetics (acute lymphoblastic leukemia);chr8:130605604-130605604;
chr8	125474726	125486817	-	ENSG00000245149	RNF139-AS1	Cholesterol, total;chr8:125478730-125478730;HDL cholesterol;chr8:125478730-125478730;LDL cholesterol;chr8:125478730-125478730;Triglycerides;chr8:125478730-125478730;Triglycerides-Blood Pressure (TG-BP);chr8:125481504-125481504;Triglycerides;chr8:125478730-

						125478730;Cholesterol, total;chr8:125478730-125478730;HDL cholesterol;chr8:125478730-125478730;LDL cholesterol;chr8:125478730-125478730;Triglycerides;chr8:125478730-125478730;Triglycerides;chr8:125478730-125478730;Triglycerides;chr8:125478730-125478730;
chr8	141530255	141539600	-	ENSG00000259758	CASC7	Breast size;chr8:141530825-141530825;

(B). LincRNA genes containing disease-associated SNPs in MCF7

Chrom	Start	End	Strand	Ensembl Name	Gene symbol	Related Trait
chr1	235093089	235105809	-	ENSG00000238005	RP11-443B7.1	Obesity-related traits;chr1:235103812-235103812;
chr10	28721937	28784742	-	ENSG00000237128	RP11-351M16.3	Preeclampsia;chr10:28738294-28738294;
chr11	86666661	86712001	+	ENSG00000246523	RP11-736K20.6	Adverse response to chemotherapy (neutropenia/leucopenia) (doxorubicin);chr11:86703240-86703240;
chr14	62182276	62217815	-	ENSG00000258667	HIF1A-AS2	Iron status biomarkers;chr14:62187980-62187980;Attention deficit hyperactivity disorder;chr14:62211585-62211585;
chr14	50394289	50428678	-	ENSG00000258946	RP11-58E21.4	Cognitive performance;chr14:50411265-50411265;
chr15	69755365	69863775	+	ENSG00000245750	RP11-279F6.1	Height;chr15:69755818-69755818;
chr15	52472290	52498071	+	ENSG00000259577	RP11-430B1.2	Metabolite levels (Pyroglutamine);chr15:52480826-52480826;
chr15	67695957	67814182	-	ENSG00000259673	IQCH-AS1	Body mass index;chr15:67788548-67788548;Obesity;chr15:67750719-67750719;Obesity;chr15:67750719-67750719;Body mass index;chr15:67788548-67788548;Restless legs syndrome;chr15:67744514-67744514;Body mass index;chr15:67794500-67794500;Restless legs syndrome;chr15:67744514-67744514;
chr17	45940708	45942556	+	ENSG00000264243	RP11-6N17.1	Progressive supranuclear palsy;chr17:45942346-45942346;
chr19	49834874	49843865	-	ENSG00000197813	CTC-301O7.4	Blood trace element (Se levels);chr19:49834979-49834979;
chr2	208125169	208135414	+	ENSG00000225064	AC007879.6	Attention deficit hyperactivity disorder;chr2:208129321-208129321;
chr2	33050510	33151760	+	ENSG00000230876	LINC00486	Height;chr2:33135271-33135271;Immune reponse to smallpox (secreted IFN-alpha);chr2:33076880-33076880;Height;chr2:33136358-33136358;
chr20	48909257	48931459	+	ENSG00000203999	RP11-290F20.1	Anger;chr20:48925063-48925063;
chr20	61798149	61812255	-	ENSG00000231977	RP5-963E22.4	β2-Glycoprotein I (β2-GPI) plasma levels;chr20:61806743-61806743;
chr3	195869507	195887761	+	ENSG00000224652	LINC00885	Bronchopulmonary dysplasia;chr3:195870036-195870036;
chr3	119813742	119855630	+	ENSG00000242622	RP11-18H7.1	HDL cholesterol;chr3:119841759-119841759;
chr4	105575031	105887950	-	ENSG00000248242	RP11-556I14.2	Lung function (forced expiratory volume in 1 second);chr4:105657597-105657597;Pulmonary function (interaction);chr4:105886950-105886950;Pulmonary function;chr4:105697983-105697983;Pulmonary function;chr4:105767747-105767747;
chr4	105828514	106041157	+	ENSG00000248373	RP11-556I14.1	Pulmonary function (interaction);chr4:105886950-105886950;
chr5	133842243	133844920	+	ENSG00000251169	AC005355.2	Amyotrophic lateral sclerosis (sporadic);chr5:133843389-133843389;
chr6	19802395	19804983	-	ENSG00000228412	RP4-625H18.2	Endometriosis;chr6:19803537-19803537;

chr6	14977019	15090234	-	ENSG00000234261	RP11-146I2.1	Multiple sclerosis (severity);chr6:15088920-15088920;
chr6	30201816	30293911	-	ENSG00000270604	HCG17	Anger;chr6:30211645-30211645;QT interval in Tripanosoma cruzi seropositivity;chr6:30256461-30256461;Autism spectrum disorder, attention deficit-hyperactivity disorder, bipolar disorder, major depressive disorder, and schizophrenia (combined);chr6:30206354-30206354;Schizophrenia;chr6:30206354-30206354;
chr6	25992890	26002003	+	ENSG00000272462	U91328.19	Iron status biomarkers;chr6:25997230-25997230;
chr8	125474726	125486817	-	ENSG00000245149	RNF139-AS1	Cholesterol, total;chr8:125478730-125478730;HDL cholesterol;chr8:125478730-125478730;LDL cholesterol;chr8:125478730-125478730;Triglycerides;chr8:125478730-125478730;Triglycerides-Blood Pressure (TG-BP);chr8:125481504-125481504;Triglycerides;chr8:125478730-125478730;Cholesterol, total;chr8:125478730-125478730;HDL cholesterol;chr8:125478730-125478730;LDL cholesterol;chr8:125478730-125478730;Triglycerides;chr8:125478730-125478730;Triglycerides;chr8:125478730-125478730;Triglycerides;chr8:125478730-125478730;
chr8	144063155	144099854	-	ENSG00000247317	RP11-273G15.2	Blood metabolite levels;chr8:144090784-144090784;Metabolic traits;chr8:144084619-144084619;
chr8	96216684	96822364	+	ENSG00000253773	KB-1047C11.2	Lung function (forced vital capacity);chr8:96251190-96251190;DNA methylation (variation);chr8:96784503-96784503;Obesity-related traits;chr8:96357406-96357406;
chr8	100008991	100025272	-	ENSG00000253948	RP11-410L14.2	Basal cell carcinoma ;chr8:100012277-100012277;Basal cell carcinoma ;chr8:100012277-100012277;
chr8	125847126	125869758	-	ENSG00000255080	RP11-1082L8.3	Metabolite levels (Dihydroxy docosatrienoic acid);chr8:125855261-125855261;
chr8	141530255	141539600	-	ENSG00000259758	CASC7	Breast size;chr8:141530825-141530825;
chr9	110182565	110193083	-	ENSG00000226825	RP11-363D24.1	Height;chr9:110183125-110183125;
chr9	84304628	84391815	+	ENSG00000233926	RP11-154D17.1	Obesity-related traits;chr9:84329510-84329510;Response to statin therapy;chr9:84307121-84307121;

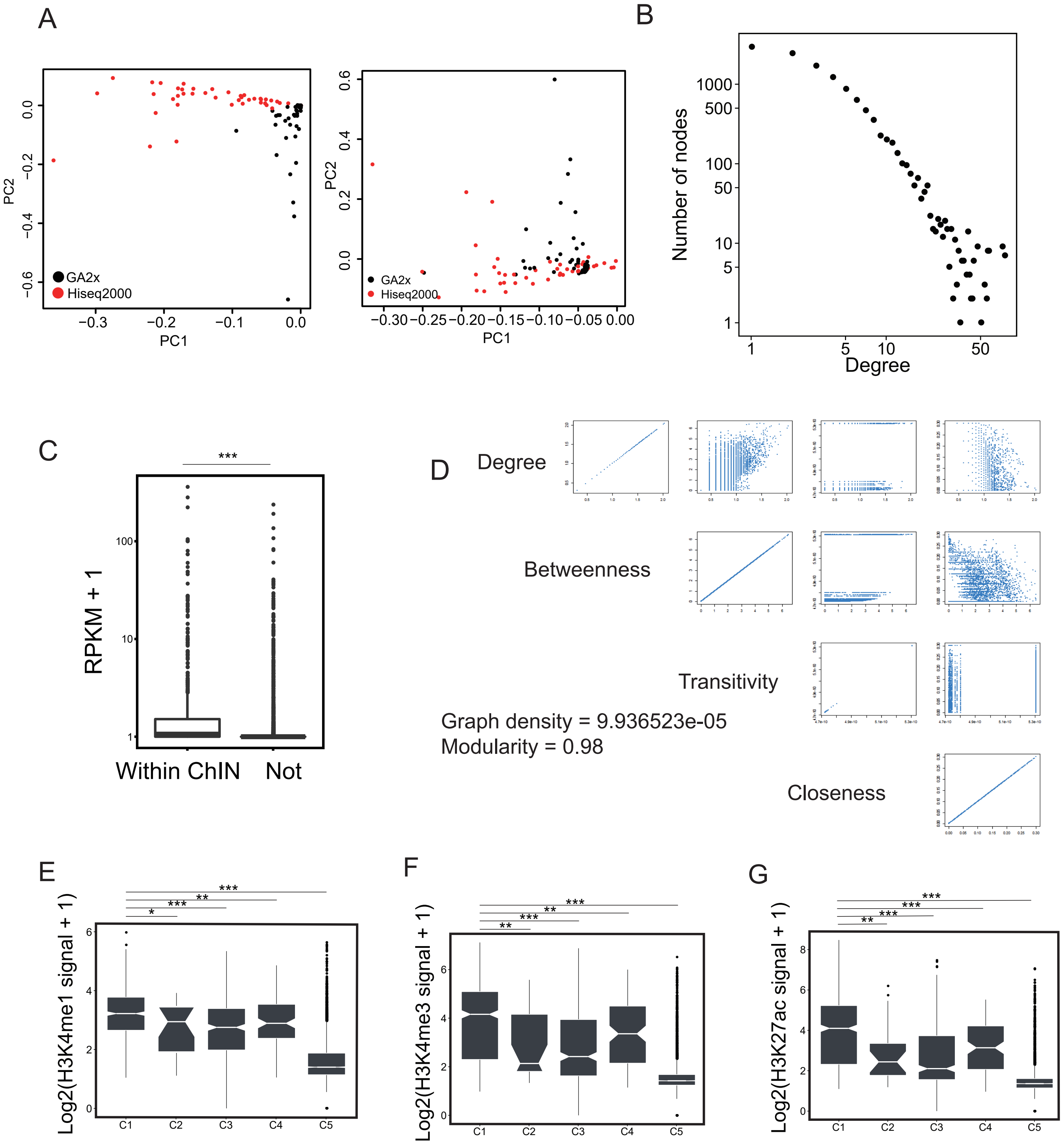
A
GA2x
Hiseq2000
PC1
PC2
B
Number of nodes
Degree
C
RPKM + 1
Within ChIN
Not
D
Degree
Betweenness
Transitivity
Closeness
Graph density = 9.936523e-05
Modularity = 0.98
E
Log2(H3K4me1 signal + 1)
F
Log2(H3K4me3 signal + 1)
G
Log2(H3K27ac signal + 1)
C1
C2
C3
C4
C5

Figure S1

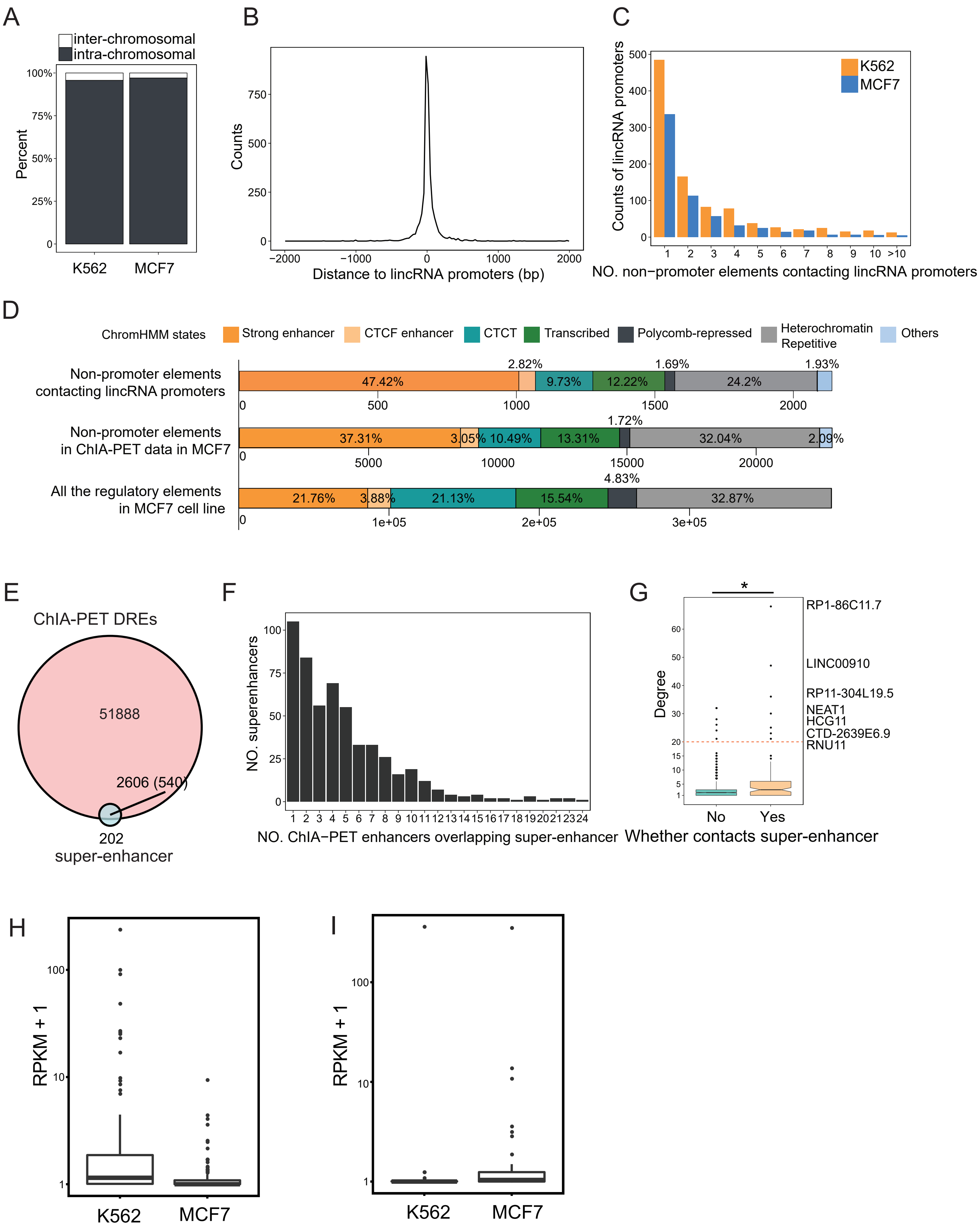
A
inter-chromosomal
intra-chromosomal
Percent
100%
75%
50%
25%
0
K562
MCF7
B
Counts
Distance to lincRNA promoters (bp)
C
Counts of lincRNA promoters
K562
MCF7
NO. non−promoter elements contacting lincRNA promoters
D
ChromHMM states
Strong enhancer
CTCF enhancer
CTCT
Transcribed
Polycomb-repressed
Heterochromatin
Repetitive
Others
Non-promoter elements
contacting lincRNA promoters
47.42%
2.82%
9.73%
12.22%
1.69%
24.2%
1.93%
Non-promoter elements
in ChIA-PET data in MCF7
37.31%
3.05%
10.49%
13.31%
1.72%
32.04%
2.09%
All the regulatory elements
in MCF7 cell line
21.76%
3.88%
21.13%
15.54%
4.83%
32.87%
E
ChIA-PET DREs
51888
2606 (540)
202
super-enhancer
F
NO. superenhancers
NO. ChIA−PET enhancers overlapping super-enhancer
G
*
Degree
RP1-86C11.7
LINC00910
RP11-304L19.5
NEAT1
HCG11
CTD-2639E6.9
RNU11
No
Yes
Whether contacts super-enhancer
H
RPKM + 1
K562
MCF7
I
RPKM + 1
K562
MCF7

Figure S2

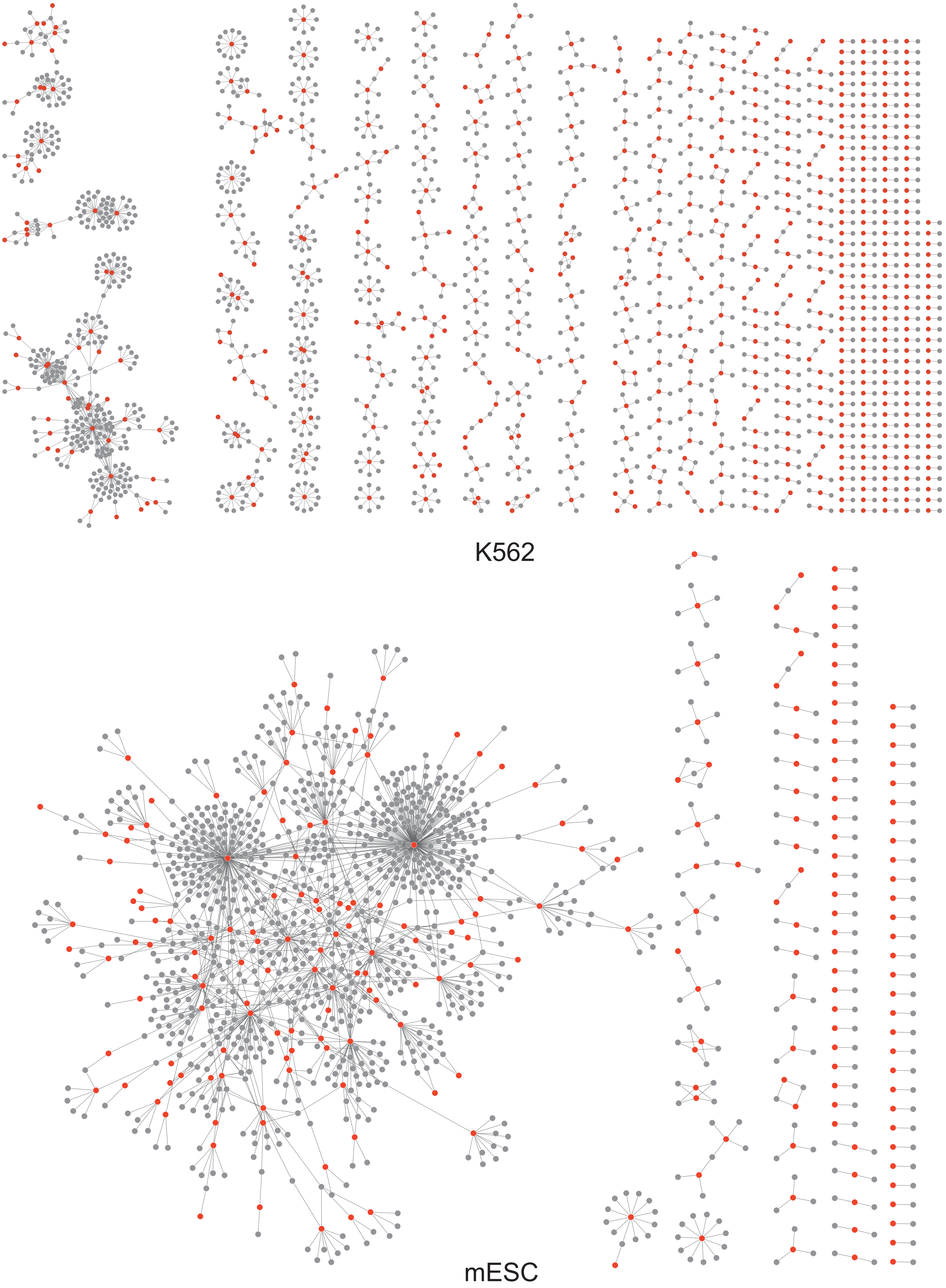

Figure S3

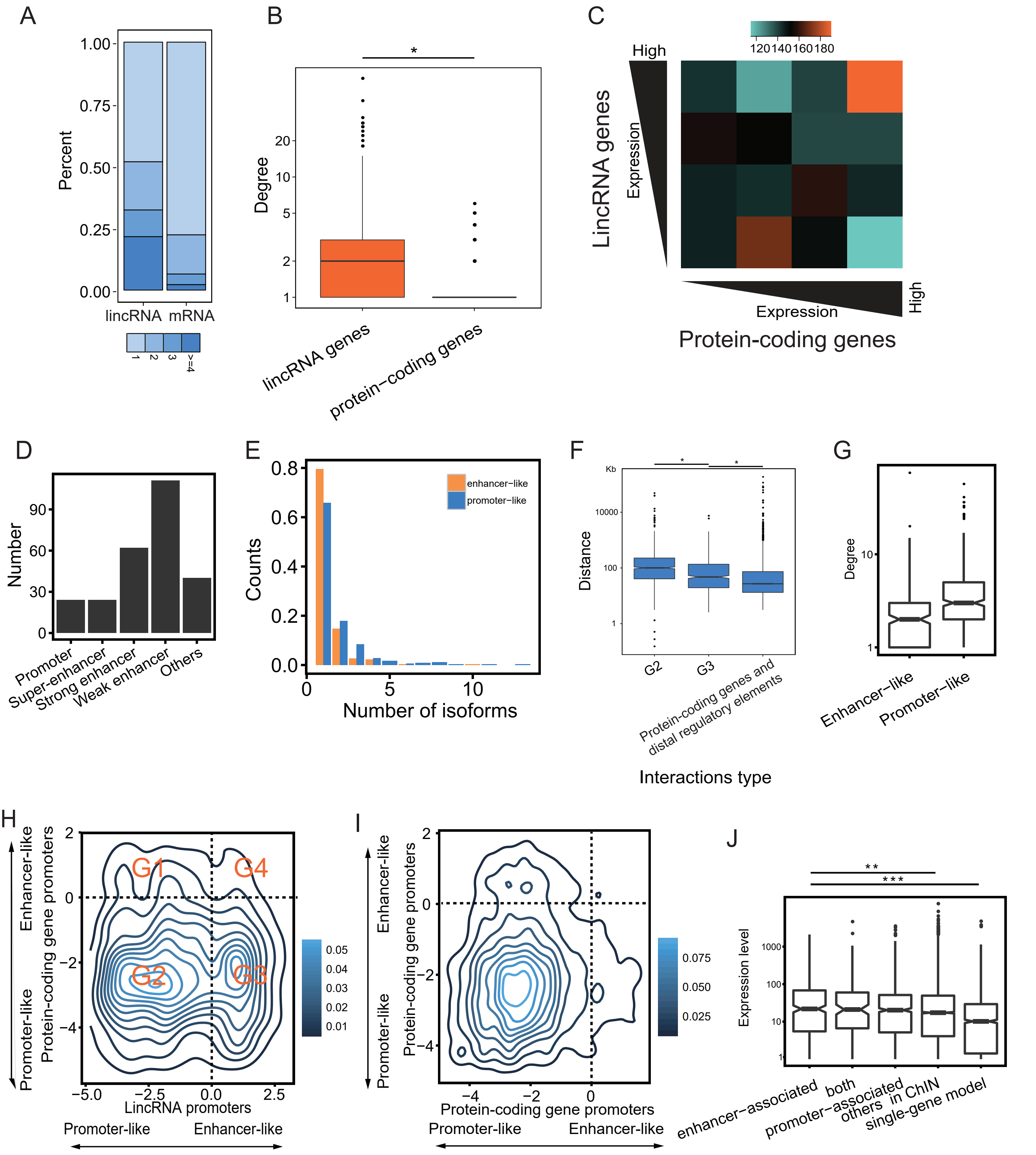
A
B
C
D
E
F
G
H
I
J
Percent
lincRNA
mRNA
Degree
lincRNA genes
protein−coding genes
High
LincRNA genes
Expression
Protein-coding genes
Number
Promoter
Super-enhancer
Strong enhancer
Weak enhancer
Others
Counts
enhancer−like
promoter−like
Number of isoforms
Distance
Interactions type
Protein-coding genes and distal regulatory elements
Enhancer−like
Promoter−like
G1
G2
G3
G4
LincRNA promoters
Protein-coding gene promoters
Expression level
enhancer−associated
both
promoter−associated
others in ChIN
single-gene model

Figure S4

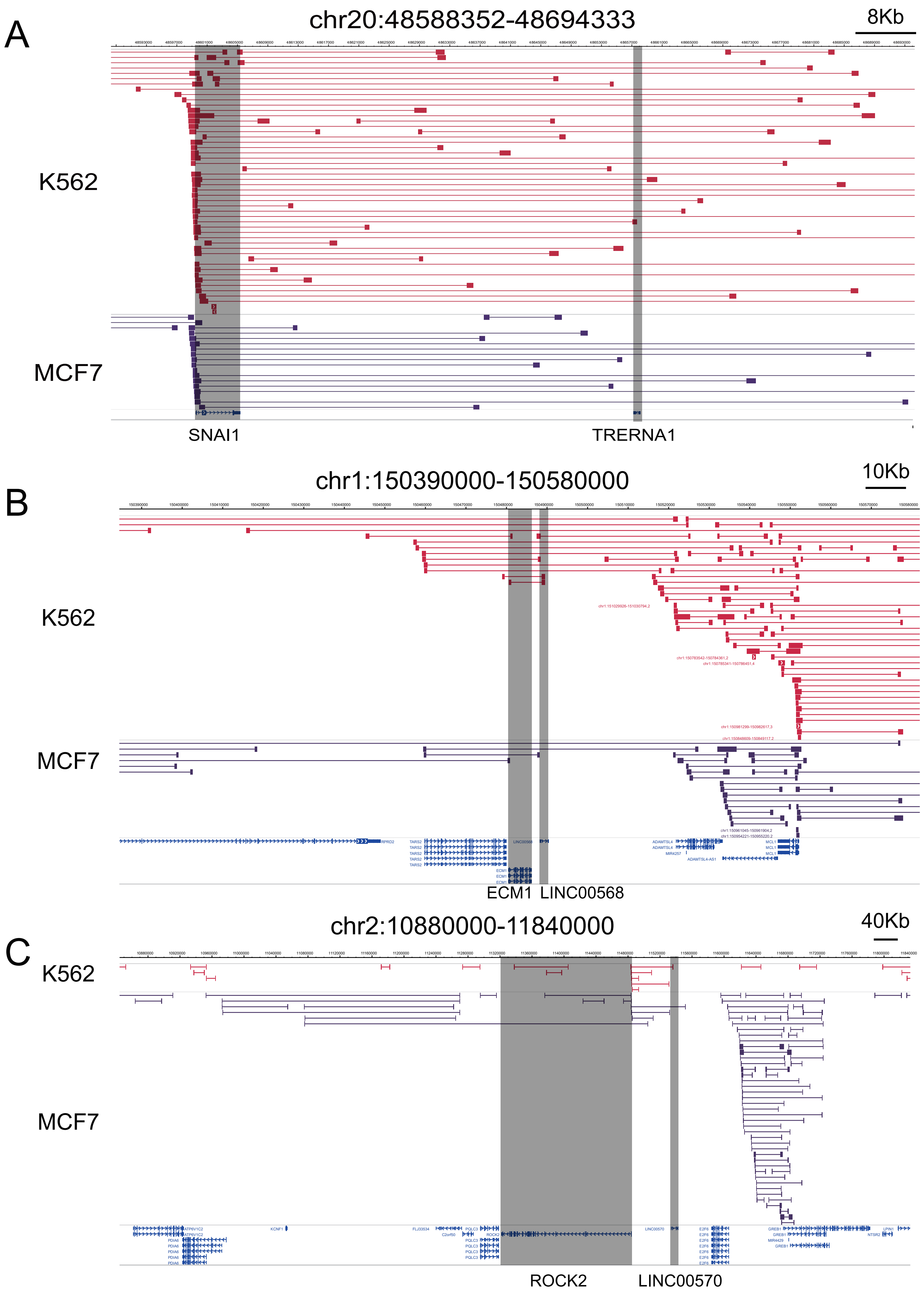
A
chr20:48588352-48694333
8Kb
K562
MCF7
SNAI1
TRERNA1
B
chr1:150390000-150580000
10Kb
K562
MCF7
ECM1
LINC00568
C
chr2:10880000-11840000
40Kb
K562
MCF7
ROCK2
LINC00570

Figure S5

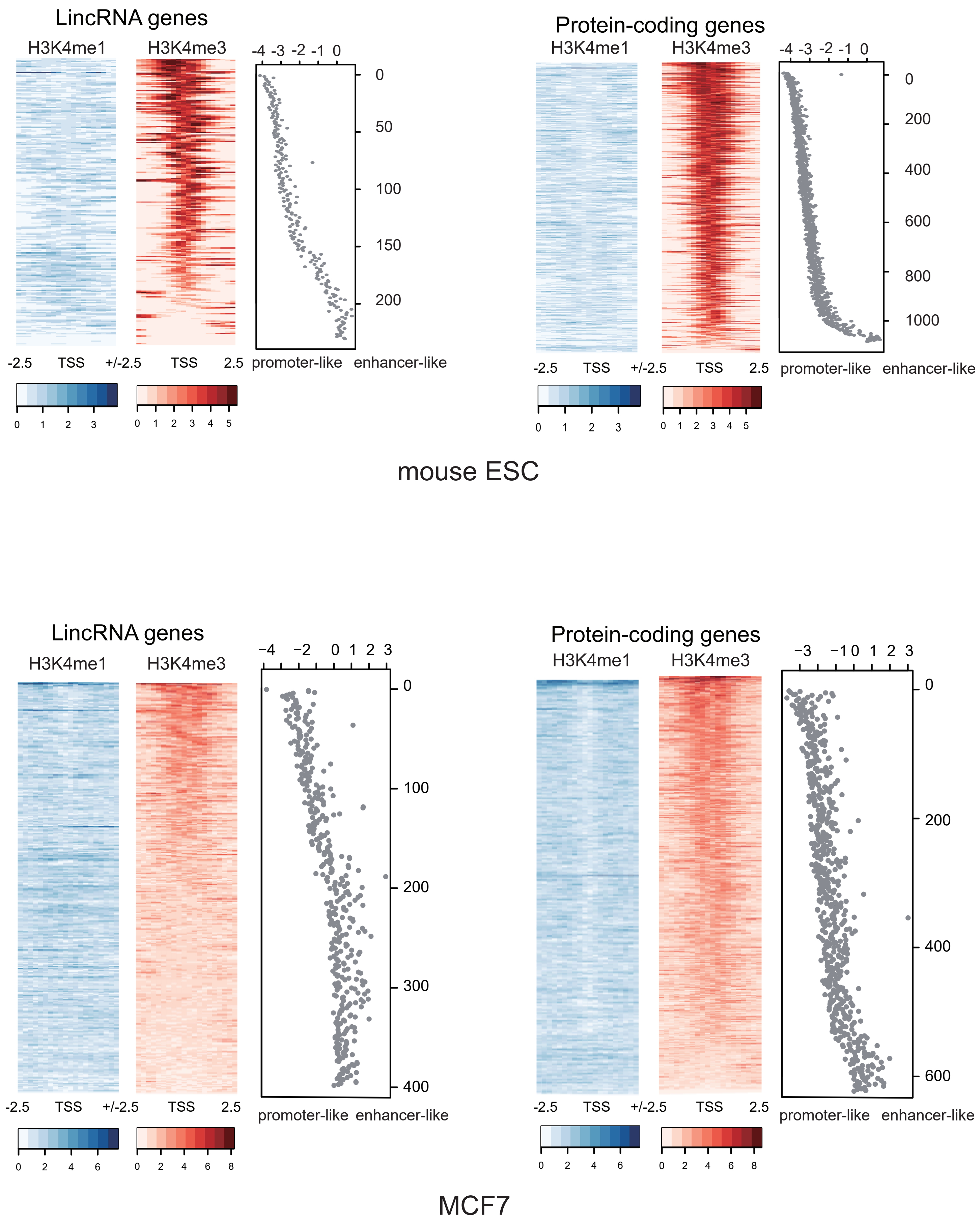
LincRNA genes
H3K4me1
H3K4me3
-4 -3 -2 -1 0
0
50
100
150
200
-2.5
TSS
+/-2.5
TSS
2.5
promoter-like
enhancer-like
0 1 2 3
0 1 2 3 4 5
Protein-coding genes
H3K4me1
H3K4me3
-4 -3 -2 -1 0
0
200
400
600
800
1000
-2.5
TSS
+/-2.5
TSS
2.5
promoter-like
enhancer-like
0 1 2 3
0 1 2 3 4 5
mouse ESC
LincRNA genes
H3K4me1
H3K4me3
−4 −2 0 1 2 3
0
100
200
300
400
-2.5
TSS
+/-2.5
TSS
2.5
promoter-like
enhancer-like
0 2 4 6
0 2 4 6 8
Protein-coding genes
H3K4me1
H3K4me3
−3 −1 0 1 2 3
0
200
400
600
-2.5
TSS
+/-2.5
TSS
2.5
promoter-like
enhancer-like
0 2 4 6
0 2 4 6 8
MCF7

Figure S6

A

GO terms with the most lincRNA genes	Cluster
detection of stimulus/ G protein coupled receptor activity/	1
structural constituent of ribosome	2
nitrogen compound catabolic process	3
cellular protein catabolic process/ regulation of muscle	4
mitochondrial membrane	5
sensory perception of chemical stimulus	6
mitosis /m phase of mitotic cell cycle/ mRNA metabolic process/ mRNA processing/RNA splicing	7
cytokine production	8
meiotic recombination	9
mitosis/ m phase of mitotic cell cycle	10

B

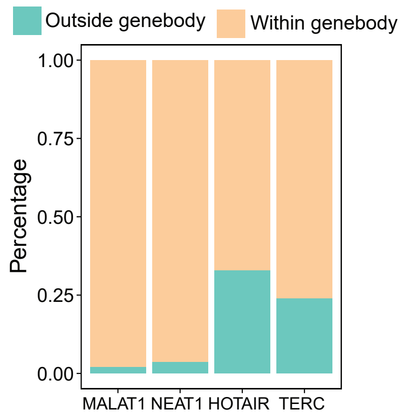

C

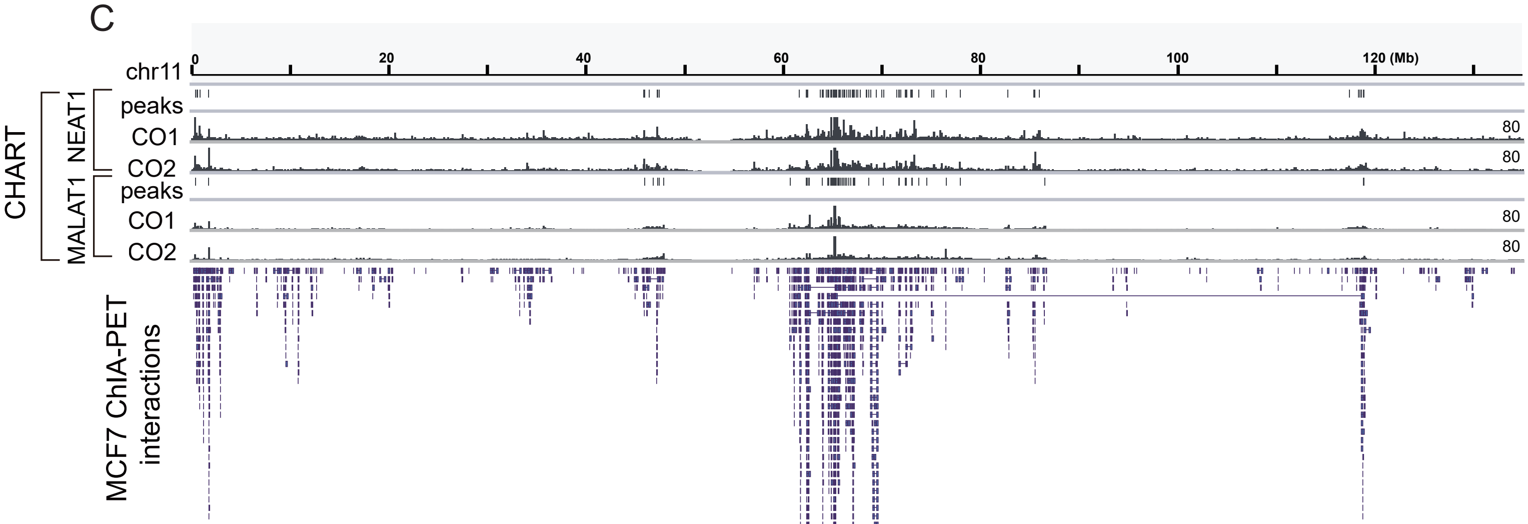

D

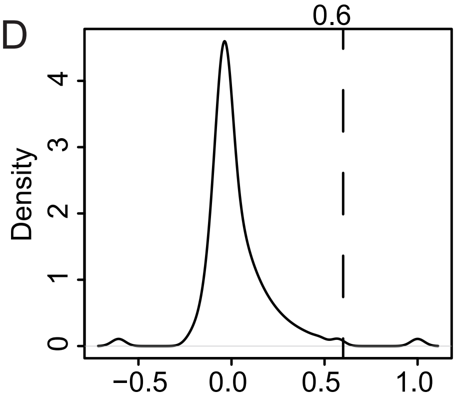

E

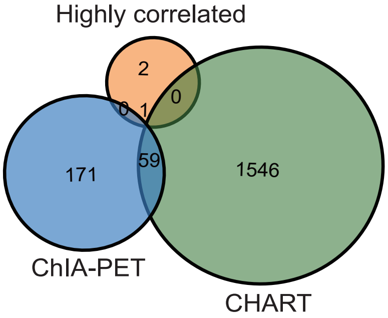

F

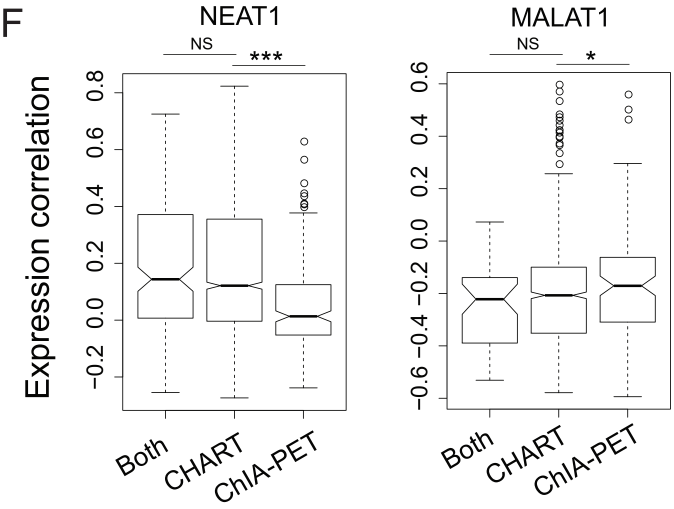

G

	NO. communities	Average genes in one community
NEAT1	439	5
MALAT1	238	4

H

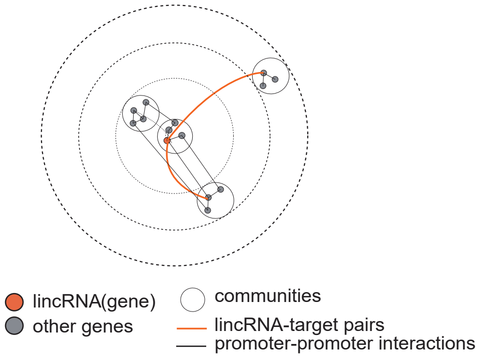

Figure S7

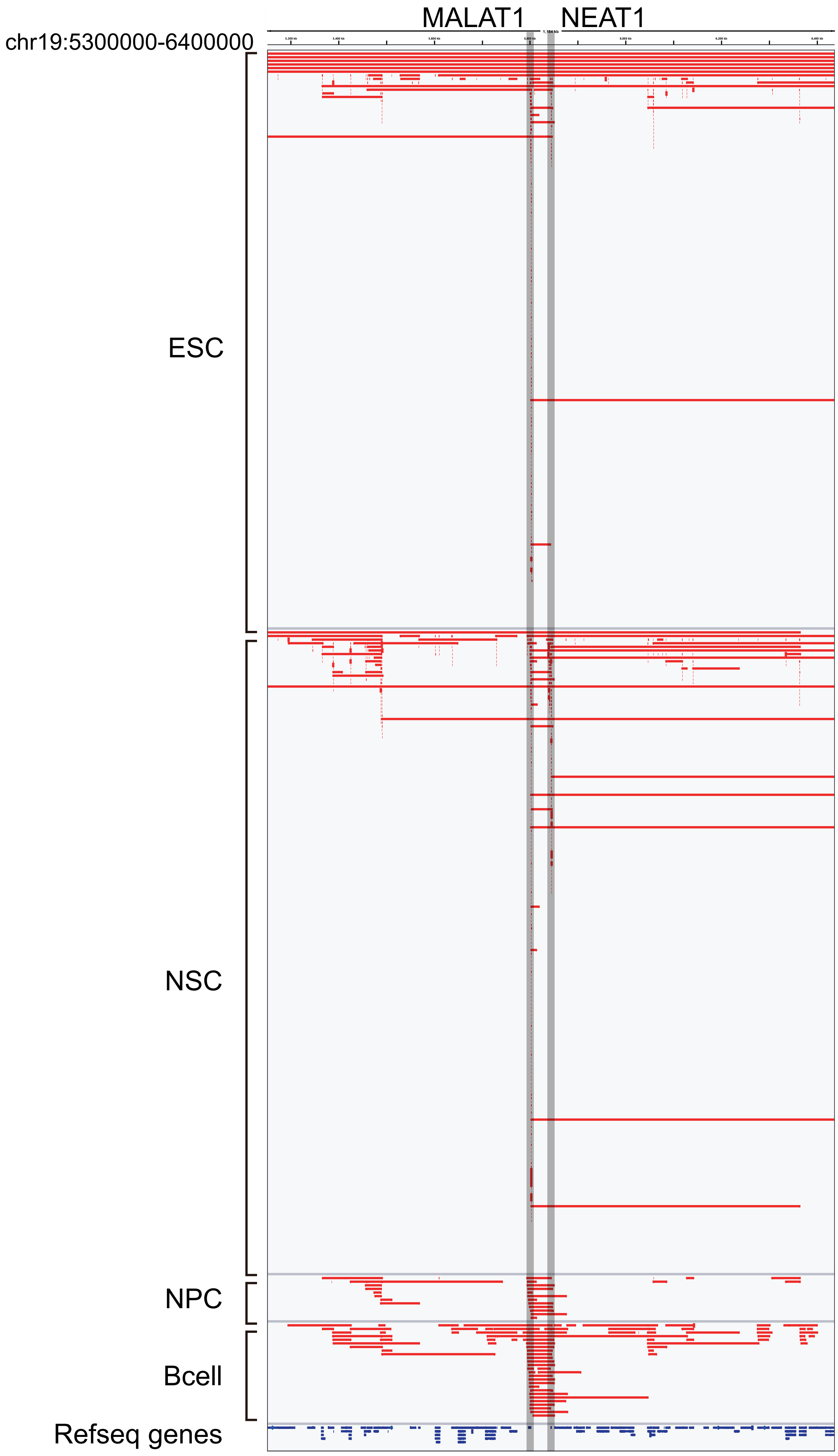
MALAT1
NEAT1
chr19:5300000-6400000
ESC
NSC
NPC
Bcell
Refseq genes

Figure S8

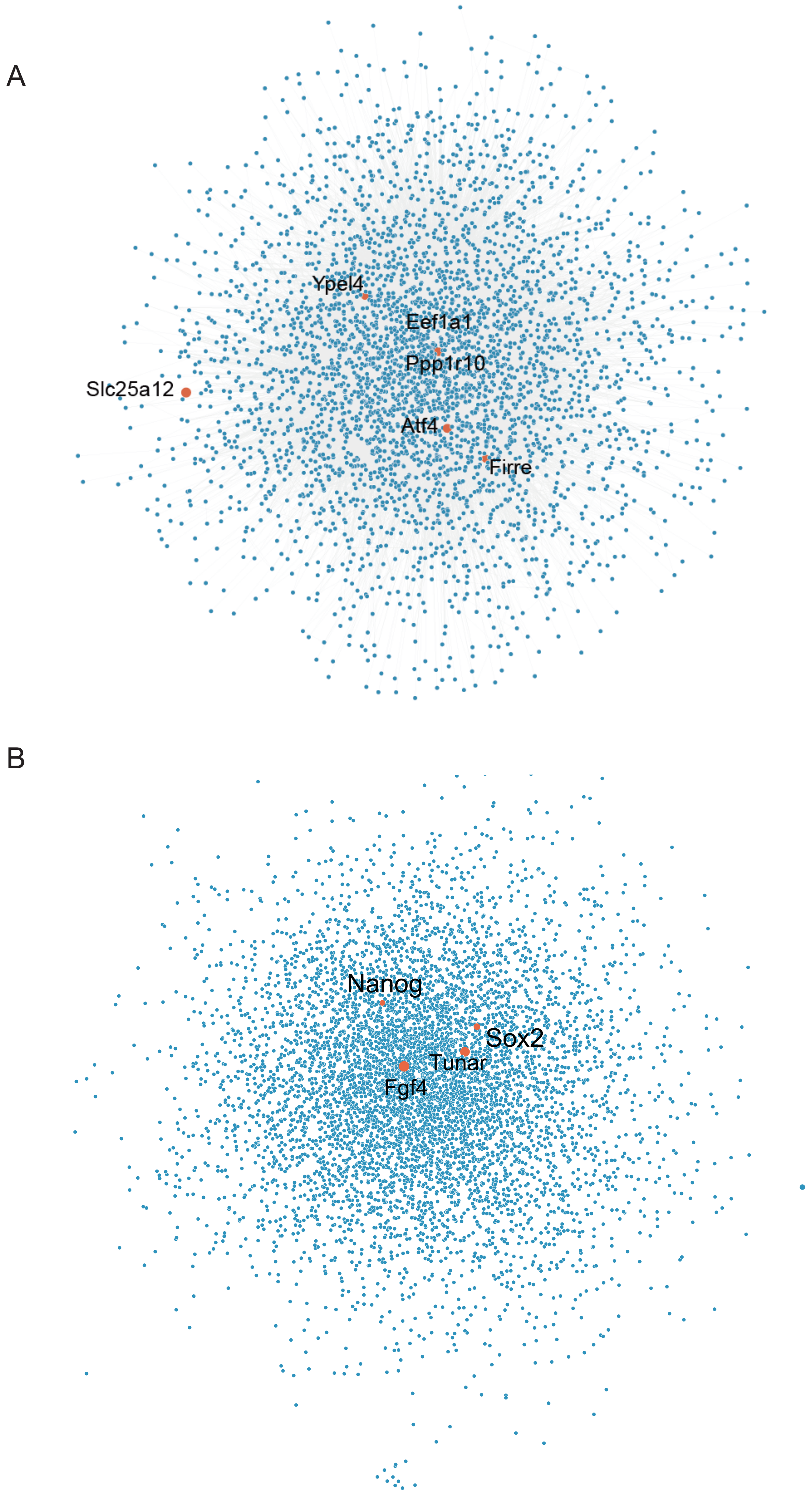
A
Ypel4
Eef1a1
Ppp1r10
Slc25a12
Atf4
Firre
B
Nanog
Sox2
Tunar
Fgf4

Figure S9

A B

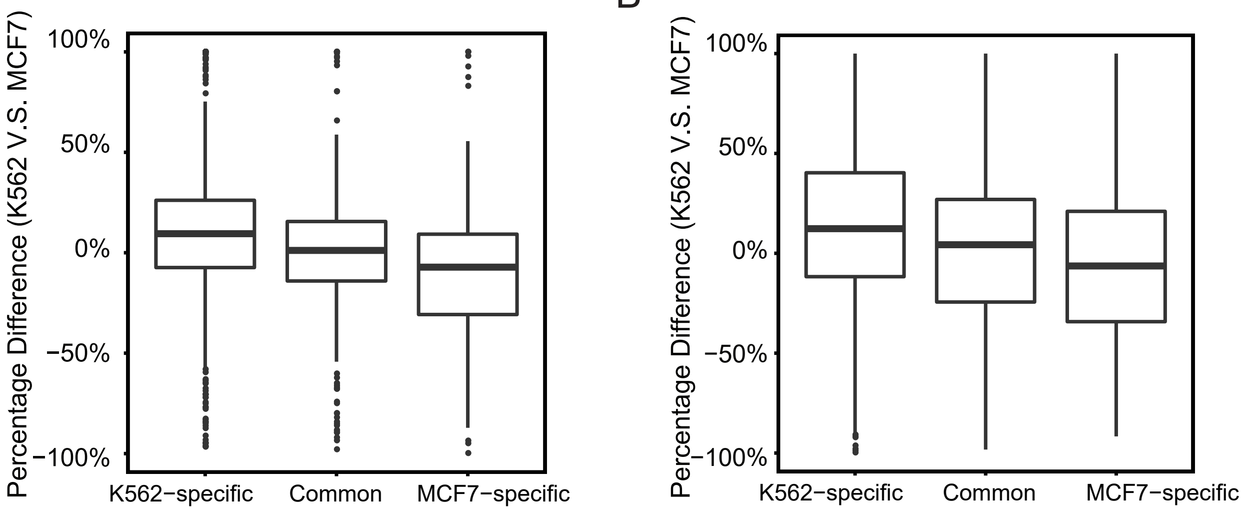

C

GO Terms (Biological Process)	Fold Change	Genes	P-value
nuclear-transcribed mRNA catabolic process, nonsense-mediated decay	4.11	15	3.3E-02
protein targeting to membrane	3.63	17	4.09E-02
nuclear-transcribed mRNA catabolic process	3.50	18	3.54E-02
translational termination	3.48	21	6.90E-03
viral life cycle	3.47	23	2.23E-03
establishment of protein localization to membrane	3.21	22	1.38E-02
translational elongation	3.21	21	2.30E-02
cellular protein complex disassembly	3.17	21	2.83E-02
translational initiation	3.13	24	7.45E-03
viral process	3.11	48	4.8E-08

D E

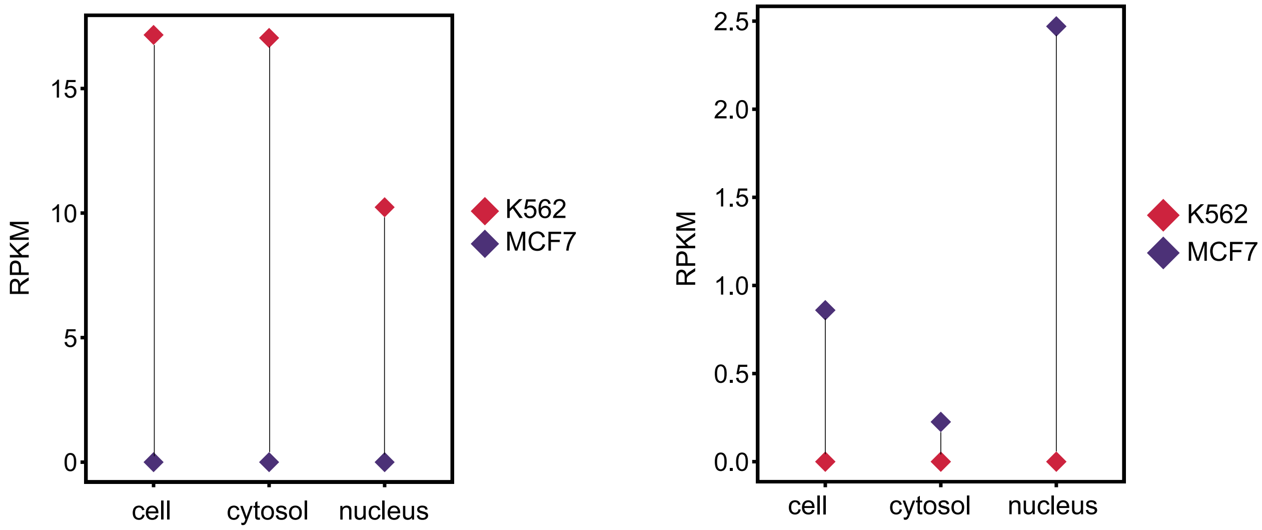

Figure S10
